# Inborn-like errors of metabolism are determinants of breast cancer risk, clinical response and survival: a study of human biochemical individuality

**DOI:** 10.18632/oncotarget.25839

**Published:** 2018-08-03

**Authors:** Ismael da Silva, Rene da Costa Vieira, Carolina Stella, Edson Loturco, André Lopes Carvalho, Carlos Veo, Cristovam Neto, Sandra M. Silva, Paulo D'Amora, Marcia Salzgeber, Delcio Matos, Celso R. Silva, Jose R. Oliveira, Iara Rabelo, Patricia Yamakawa, Rui Maciel, Rosa Biscolla, Maria Chiamolera, Renato Fraietta, Felipe Reis, Marcelo Mori, Dirce Marchioni, Antonio Carioca, Gustavo Maciel, Renato Tomioka, Edmund Baracat, Clovis Silva, Celso Granato, Ricardo Diaz, Bruno Scarpellini, Daniel Egle, Heidi Fiegl, Irmgard Himmel, Christina Troi, Robert Nagourney

**Affiliations:** ^1^ Gynecology Department, College of Medicine of the Federal University of São Paulo (EPM-UNIFESP), São Paulo, Brazil; ^2^ Fleury Laboratories, São Paulo, Brazil; ^3^ Department of Surgery, Surgical Gastroenterology Division, College of Medicine of the Federal University of São Paulo (EPM-UNIFESP), São Paulo, Brazil; ^4^ Clinical and Experimental Oncology Department, Hematology and Hemotherapy Division, College of Medicine of the Federal University of São Paulo (EPM-UNIFESP), São Paulo, Brazil; ^5^ Department of Medicine, Endocrinology Division, College of Medicine of the Federal University of São Paulo (EPM-UNIFESP), São Paulo, Brazil; ^6^ Department of Surgery, Urology Unit, Human Reproduction Division, College of Medicine of the Federal University of São Paulo (EPM-UNIFESP), São Paulo, Brazil; ^7^ Biophysics Department, College of Medicine of the Federal University of São Paulo (EPM-UNIFESP), São Paulo, Brazil; ^8^ Barretos Cancer Hospital (HCB), Barretos, Brazil; ^9^ Department of Biochemistry and Tissue Biology, State University of Campinas (UNICAMP), Campinas, Brazil; ^10^ Nutrition Department, School of Public Health, University of São Paulo School of Medicine (FMUSP), São Paulo, Brazil; ^11^ Department of Obstetrics and Gynecology, University of São Paulo School of Medicine (HCFMUSP), São Paulo, Brazil; ^12^ Department of Pediatrics, Children's Hospital, University of São Paulo School of Medicine (HCFMUSP), São Paulo, Brazil; ^13^ Retrovirology Laboratory, Infectious Diseases Unit, Medicine Department, College of Medicine of the Federal University of São Paulo (EPM-UNIFESP), São Paulo, Brazil; ^14^ Department of Obstetrics and Gynecology, Medical University of Innsbruck, Innsbruck, Austria; ^15^ Department of Gynecology, Meran Hospital, Meran, Italy; ^16^ Department of Gynecology, Brixen Hospital, Brixen, Italy; ^17^ Department of Obstetrics and Gynecology, Gynecological Oncology Unit, University of California Irvine (UCI), California, USA

**Keywords:** breast cancer, metabolism, prognosis, response, survival

## Abstract

Breast cancer remains a leading cause of morbidity and mortality worldwide yet methods for early detection remain elusive. We describe the discovery and validation of biochemical signatures measured by mass spectrometry, performed upon blood samples from patients and controls that accurately identify (>95%) the presence of clinical breast cancer. Targeted quantitative MS/MS conducted upon 1225 individuals, including patients with breast and other cancers, normal controls as well as individuals with a variety of metabolic disorders provide a biochemical phenotype that accurately identifies the presence of breast cancer and predicts response and survival following the administration of neoadjuvant chemotherapy. The metabolic changes identified are consistent with inborn-like errors of metabolism and define a continuum from normal controls to elevated risk to invasive breast cancer. Similar results were observed in other adenocarcinomas but were not found in squamous cell cancers or hematologic neoplasms. The findings describe a new early detection platform for breast cancer and support a role for pre-existing, inborn-like errors of metabolism in the process of breast carcinogenesis that may also extend to other glandular malignancies.

Statement of Significance: Findings provide a powerful tool for early detection and the assessment of prognosis in breast cancer and define a novel concept of breast carcinogenesis that characterizes malignant transformation as the clinical manifestation of underlying metabolic insufficiencies.

## INTRODUCTION

Breast cancer remains a leading cause of morbidity and mortality throughout the world [1, 2]. Earlier diagnosis through the application of mammography and magnetic resonance imaging has improved the detection of smaller volume disease providing physicians the opportunity to intervene at earlier stages when the cancers are most curable [3].

The advent of molecular technologies, widely applied in prognostic determinations, have evolved into diagnostic tools that utilize circulating tumors cells and cell free DNA for earlier detection, prognosis and where applicable response prediction. Numerous clinical trials are now exploring the clinical utility of these approaches [4, 5].

We now recognize that human cancers evolve in an environment of metabolic stress. Rapidly proliferating tumor cells deprived of adequate oxygen, nutrients, hormones and growth factors up-regulate pathways that address these deficiencies to overcome hypoxia (HIF), vascular insufficiency (VEGF), growth factor deprivation (EGFR, HER2) and the loss of hormonal support (ER, PR, AR) all to enhance survival and proliferation [6].

Many oncogenes are now known to regulate metabolic pathways that are critical for cell survival in the inhospitable tumor micro-environment, where oxygen and nutrient sources are highly limited. Indeed RAS, PI3K, TP53 and MYC among others are now recognized to be important metabolic regulators whose functions are fundamental for tumor cell survival [7].

Based upon the growing recognition that cancer cells differ from their normal counterparts in their use of nutrients, synthesis of biomolecules and generation of energy, we applied quantitative mass spectrometry to the blood and tissue of patients with breast cancer and compared the results with those observed in normal controls. To explore commonalties, we extended these studies to include other cancers of glandular and non-glandular ancestries and to non-malignant disease states associated with metabolic stress including poly cystic ovary syndrome and advanced metabolic syndrome.

The findings led to a murine model of insulin/glucose mediation of metabolic stress and finally to an exploration of the secretome of human embryos prior to implantation to examine the “stemness” of the signals observed.

## RESULTS

### Breast cancer identification through blood biochemical phenotyping

**Figure 1:**
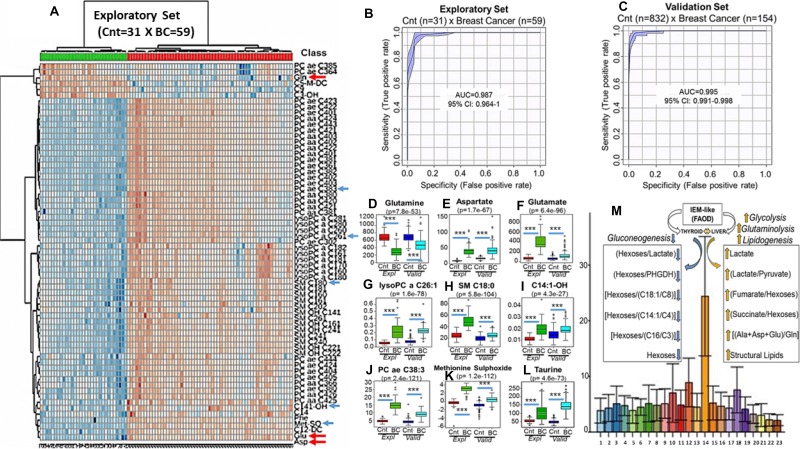
Breast cancer discriminative performance of the top 50 individual metabolites quantified in blood (μmol/L), during exploratory set, using unsupervized clustering analysis with heatmap (**A**). Arrows are pointing to metabolites whose concentrations in blood (μmol/L) were analyzed by ANOVA during exploratory (Expl) (Red and Green Bars) and confirmed after validation (Valid) set (Dark and Light Blue Bars). The first red arrow at the top (a) show glutamine (Gln), the most abundant amino acid in healthy population (Cnt), whose concentrations, however, became very low in blood of breast cancer women (**B**, **C**) (**D**). On the other hand, the two red arrows at the bottom (A) are pointing to glutamate (Glu) and aspartate (Asp) whose concentrations are high in the blood of the same patients (**E** and **F**). This description completely fullfils the concept of “Glutaminolysis” where glutamine is consumed and transformed in glutamate and aspartate. The increased concentrations of sphyngomielins (SM C18:0) (**G**) and ether lipids (PC ae C38:3) (**H**) are suggestive that a systemic metabolic shift favoring biosynthesis is predominant in cancer patients. Accumulations, in blood, of acylcarnitines and lipids containing very-long chain fatty acids (C14:1-OH) (**I**) (lysoPC a C26:1) (**J**) are common metabolic features of mitochondrial and peroxisomal fatty acids oxidation deficiencies (FAOD) that are, usually followed, by disturbances in ReDOX homeostasis with elevations in oxidative stress and consequent damage to proteins as demonstrated by significant elevations in methionine sulphoxide residues (Met-SO) (**K**). Elevations in taurine (**L**), as will be demonstrated ahead, are directly related to increases in blood levels of oncometabolites succinate and fumarate. Figure 1A and 1B are showing the breast cancer discriminative performance during exploratory (A) and validation (B) sets using the equation {PC aa C36:6/[(Xle/Phe)/Tau]}/C102 and the lipid PC aa C28:1 whose absolute concentrations in blood were applied to multivariate ROC curve analysis. Increasing values generated by this metabolic signature were able to accurately segregate breast cancer from controls either during training [AUC = 0.987 (95% CI: 0.964-1), sensitivity = 96.72%, specificity = 96.78%, positive predictive value = 98.33% negative predictive value = 93.94%, average accuracy (100-fold cross validations) = 0.95 and predictive accuracy statistics (1000 permutations) = *p* < 2.04e-05] or validation sets [AUC = 0.995 (95% CI: 0.991-0.998), sensitivity = 98.09%, specificity = 96.18%, positive predictive value = 82.35%, negative predictive value = 99.64%, average accuracy (100-fold cross validations) = 0.96 and predictive accuracy statistics (1000 permutations) = *p* < 1.28e-06]. (**M**) depicts the positive (orange arrows) and negative (blue arrows) correlations among the increasing values of our ratio (Y-Axis) and the oncometabolites succinate, fumarate, lactate and hexoses as well as glutaminolysis (Ala+Asp+Glu/Gln) and structural lipids measured in different metabolic groups (X-Axis): (1)-grade zero metabolic syndrome; (2)-grade 1 metabolic syndrome; (3)-grade 2 metabolic syndrome; (4)-grade 3 metabolic syndrome; (5)-grade 4 metabolic syndrome; (6)-grade 5 metabolic syndrome; (7)-High Risk (RR 1.4) Breast Cancer; (8)-High Risk (RR 1.6) Breast Cancer; (9)-High Risk (RR 1.8) Breast Cancer; (10)-Polycistic Ovary Syndrome; (11)-Colon Cancer; (12)-Lung Cancer; (13)-Hepatocarcinoma; (14)-Breast Cancer; (15)-Hematological Malignancies (Baseline); (16)-Hematological Malignancies (Transplant); (17)-Hematological Malignancies (Engraftment); (18)-Head and Neck Cancer; (19)-Cirrhosis; (20)-Juvenile Arthritis; (21)-Autoimmune Hemolityc Anemia; (22)-Paroxysmal Nocturnal Haemoglobinuria and (23)-Human Immunodeficiency Virus. ^***^ Indicates *p* < 0.001.

The search for metabolic intermediates, the blood concentrations of which (μM/L) could be utilized as breast cancer biomarkers led to the assembly of an exploratory data set that compared plasma samples from women at low risk of breast cancer (*n* = 31) with plasma samples from patients with treatment-naive stage III (T3N2M0) invasive disease (*n* = 59). Targeted quantitative MS/MS analysis [8] coupled with unsupervised clustering analysis (Online methods) identified clear metabolic differences between cases and controls (Figure 1A). Validation was then undertaken (statistical power = 0.8) that compared 169 population-based control samples, against results obtained in 154 cases from an independent and earlier reported disease cohort the “Risk Prediction of Breast Cancer Metastasis Study” (Italy and Austria) (Supplementary Information) (Figure 1D–1L).

Results demonstrated that breast cancer women exhibited at least one up- or down-regulated metabolite from amongst 5 of the principal 7 classes of metabolites that we quantified in blood (Supplementary Table 1A–1C) as exemplified in Figure 1A (arrows). Figure 1D–1L show the statistical analysis depicting the individual validation (dark and light blue bars) of nine of these metabolites, originally identified in the exploratory phase (red and green bars) including glutamine (Gln), aspartate (Asp), glutamate (Glu), lysophosphatidylcholine acyl C26:1 (lysoPC a C26:1), Sphingomyelin C18:0 (SM C18:0), 3-Hydroxytetradecenoylcarnitine (C14:1-OH), phosphatidylcholine acyl-alkyl C38:3 (PC ae C38:3), methionine sulfoxide (Met-SO) and taurine.

Among the observations in both, the exploratory and the validation sets, was the finding that glutamine concentrations in the cancer patients were reduced to nearly 1/8 of the levels observed in the normal population (~800 μM/L) (*p* = 7.8e-53, FDR = 2.7e-52) (Figure 1D) while blood concentrations of aspartate (*p* = 1.7e-67, FDR = 8.3e-67) (Figure 1E) and glutamate (*p* = 6.4e-96, FDR = 6.2e-95) (Figure 1F) were nearly 10 fold higher than the normal ranges of 0–5 μM/L and 40 μM/L, respectively.

As glutamine consumption associated with parallel increases in glutamate and aspartate (Figure 1A red arrows) is considered a hallmark of MYC-driven “glutaminolysis” [9], these findings led an examination of other MYC-associated phenomena to interrogate the observations.

### Blood quantification of MYC activity and its connection to metabolic syndrome, breast cancer risk, response and survival

Hepatic glutamine (Gln) metabolism regulates the level of amino acids in the circulation and Glutamate (GLU) through its role in numerous trans-deamination reactions is central to this process [9].

As MYC activation is associated with measureable changes in blood levels of specific metabolites including glutamine, glutamate, the ratios thereof and others, we used targeted quantitative MS/MS to evaluate (μM/L) these intermediates as surrogate markers for MYC activation. We then assembled metabolite ratios measured directly in blood to serve as “proxies” for MYC-coordinated metabolic functions (Online methods).

**Figure 2:**
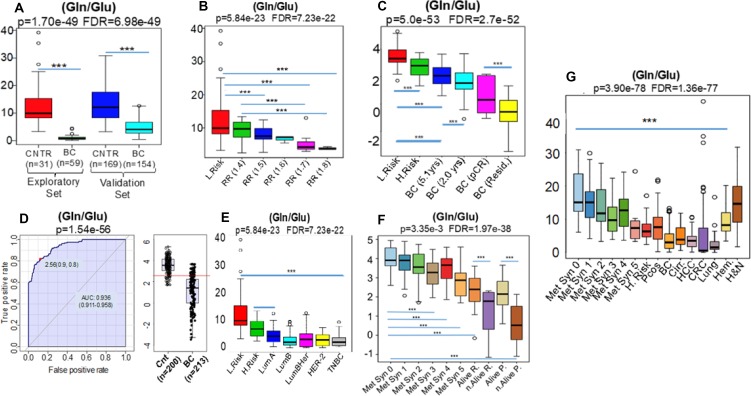
The lower values generated by the ratio (Gln/Glu), a negative surrogate for glutamine metabolism, were: i- able to discriminate breast cancer cases (BC) from controls (CNTR) (**A**, and **D**) independent of intrinsic subtypes (**E**); ii- inversely correlated with increased relative risk (RR) of breast cancer development [RR 1.4 (*n* = 8) -> 1.5 (*n* = 11) -> 1.6 (*n* = 3) -> 1.7 (*n* = 8) -> 1.8 (*n* = 3)] (**B**) and iii- inversely correlated with progressive stages (2) of metabolic syndrome Met Syn 0 (*n* = 18) -> Met Syn 1 (*n* = 46) -> Met Syn 2 (*n* = 32) -> Met Syn 3 (*n* = 41) -> Met Syn 4 (*n* = 20) -> Met Syn 5 (*n* = 9) and also to women with increased chances of death detected initially in the retrospective arm [Alive R. (*n* = 24) × not Alive R. (*n* = 8)] with further validation in the prospective setting [Alive P. (*n* = 67) X not Alive P. (*n* = 7)] of the european cohort (**C**). This ratio, besides inverse correlation to breast cancer risk (Low Risk *n* = 31 X High Risk *n* = 33) was also inversely correlated with the highest risk of 5 yrs-mortality among patients harboring tumors of 1.0 cm [BC (2.0 yrs) (*n* = 3) × BC (5.1 yrs) (*n* = 32)] as well as with the absence of pathologic complete remission (BC pCR, *n* = 7) when compared to women exhibiting residual disease (BC Resid, *n* = 52) after neo-adjuvant chemotherapy (**F**). In (**G**) results demonstrated enhanced glutamine consumption not only in plasma of patients harboring different adenocarcinomas such as: breast cancer (BC *n* = 213), colon cancer (CRC *n* = 85), Lung *n* = 23 and liver (HCC *n* = 30), but also in cancer-free subjects with cirrhosis (Cirr. *n* = 30), polycystic ovary syndrome (PCOS *n* = 49), high risk of breast cancer development (H.Risk *n* = 33) and late stage metabolic syndrome (Met Syn 3, 4 and 5). Head and neck squamous cells carcinomas (H&N *n* = 57) and hematological malignancies (Hem *n* = 65), apparently, did not share similar glutaminolytic profiles, particularly H&N tumors. ^***^ Indicates *p* < 0.001.

In agreement with our hypothesis the Gln/Glu ratio, a negative surrogate for glutamine metabolism, i- discriminated breast cancer cases from controls (Figure 2A, and 2D); ii- inversely correlated (Correlation = −0.54, *p* = 3.67e-6 FDR = 3.06e-5) with elevated breast cancer risk (Figure 2B) iii. correlated with the risk of 5-year mortality in pathological stage I patients and iv-inversely correlated with the failure to achieve pathologic complete remission (pCR) after neo-adjuvant chemotherapy (NAC) (Correlation = −0.81, *p* = 1.15e-81, FDR = 2.13e-80) (Figure 2C).

Parallel analyses found that the Gln/Glu ratio inversely correlates with i- late stage metabolic syndrome and with ii- increased chance of death in both the retrospective and prospective arms of the European cohort (Correlation = −0.68, *p* = 2.30e-38, FDR = 1.59e-37) (Figure 2F). Where applicable, *T* Test, ANOVA and posthoc analysis are highlighted by ^*^ in all figures.

Theoretically, changes in glutamine consumption, reflected by the Gln/Glu ratio could provide a metabolic link between breast cancer initiation and diabetes, reflective of a systemic metabolic reprogramming from glucose to glutamine as the preferred source of precursors for biosynthetic reactions and cellular energy [9].

We found the same changes in the Gln/Glu ratio in nearly 100% of breast cancer patients, independent of intrinsic subtype (Figure 2A, and 2E). These breast cancer patients revealed systemic MYC-associated biochemical shifts, previously described *in vitro* [9], associated with glutamine utilization over glucose for the synthesis of structural phospholipids, as measured by the ratios (Structural Lipids/Gln) and (Structural Lipids/Hexoses) respectively (Supplementary Figure 1D and 1E). The MYC signatures in breast cancer patients and their similarity to diabetes mellitus raised the question whether metabolic re-programming might be identified through the measurement of other bio-chemical intermediates.

Similar changes in glutamine consumption had previously been reported in the Framingham Heart Study where the follow-up of more than 1000 participants showed that lower Gln/Glu ratios inversely correlated with insulin resistance and the risk of diabetes [10].

### Assembling biochemical equations for breast cancer identification by incorporating elevations in oncometabolites

To examine breast cancer against other disease states, we compared our results with those obtained from other cancers (30 liver; 23 lung; 85 colon; 58 head & neck and 65 hematologic) and from individuals with various metabolic conditions including late stages of metabolic syndrome [2] (*n* = 70), HCV-induced cirrhosis (*n* = 30); hyperthyroidism (*n* = 8); hypothyroidism (*n* = 8); HIV infection (*n* = 18); polycystic ovary syndrome (*n* = 49); auto immune disease (*n* = 86) and with those from women at elevated risk for breast cancer (*n* = 33).

We measured biochemically-active metabolites, that had previously been described in large metabolomics and genome-wide association studies [11, 12] (Online methods) to examine established single metabolite and metabolite ratios related to: i- liver function (Val/Phe, Xle/Phe), ii- lipid desaturase activity (PC aa C36:6) and iii- serine palmitoyltransferase (SPTLC3) activity (PC aa C28:1 and C10:2). These measures were used to develop algorithms for the interrogation of our data sets (Online Methods).

Results, as multivariate Receiver Operator Curve (ROC) analyses, using the equation {[PC aa 36:6/[(Val/Phe)/Taurine]/C10:2} and the lipid PC aa C28:1, were found to segregate breast cancer from controls, irrespective of stage (I to III) and intrinsic subtypes, in both the exploratory [AUC = 0.987 (95% CI: 0.964-1), sensitivity = 96.72%, specificity = 96.78%, positive predictive value = 98.33%, negative predictive value = 93.94%, average accuracy (100-fold cross validations) = 0.95 and predictive accuracy statistics (1000 permutations) = *p* < 2.04e-05] and validation sets [AUC = 0.995 (95% CI: 0.991–0.998), sensitivity = 98.09%, specificity = 96.18%, positive predictive value = 82.35%, negative predictive value = 99.64%, average accuracy (100-fold cross validations) = 0.96 and predictive accuracy statistics (1000 permutations) = *p* < 1.28e-06] (Figure 1B and 1C).

**Figure 3:**
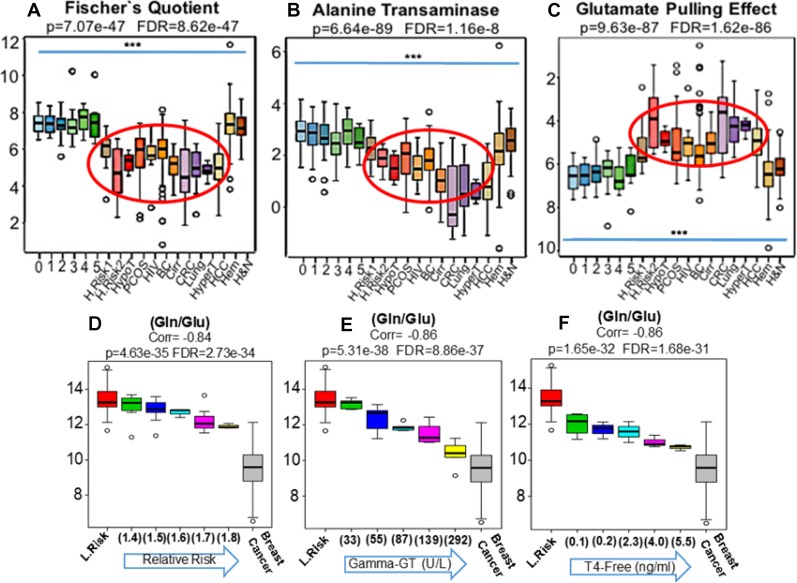
The MYC-coordinated and malignancy-associated increase in glutamate production, after *in vitro* shortages of glucose, was previously described as the “glutamate pulling effect” [11] The ratio (Glu/Hexoses) was adopted here as a proxy for this metabolic shift that, in fact, was clearly replicated in the blood (Red eclipse) of patients harboring adenocarcinomas (BC, CRC, Lung and HCC) as well as in women at higher risks of breast cancer development (R.R. = 1.5 H.Risk 1) and (R.R. = 1.8 H.Risk 2) and in individuals with PCOS (C). Of note, neither in the population-based controls depicting progressive metabolic syndrome (0 to 5) or in patients harboring non-glandular tumors such as leukemias, myelomas and lymphomas (Hem) as well H&N squamous cells carcinomas revealed significant changes in the ratio (Glu/Hexoses) (C). Since the results generated by the Fischer's quotient (**A**) were persistently suggesting liver dysfunctions in patients harboring glandular malignancies, we also compared our findings to well established conditions of liver dysfunctions such as cancer-free patients with HCV-induced cirrhosis (Cirr), patients with hypo (HypoT) and hyperthyroidism (HyperT), as thyroid dysfunction is very frequently associated with liver metabolic abnormalities as well as to increased risks of breast cancer [23–25]. Similarly, we also analyzed HIV patients due to increased risks of cancer development and because of the direct HIV influence on liver function (26). Results revealed concordance between the blood phenotypic profiles of cancer-free patients with cirrhosis, thyroid dysfunction and HIV infection with study participants at elevated relative risks of cancer, those with polycystic ovary syndrome (PCOS) and patients harboring glandular malignancies (**A**–**C** Red ellipses). To explore in more details the relations among malignancy, thyroid and liver function, we further divided our cancer-free groups according to: i- increasing relative risks of breast cancer (from 1.4 to 1.8) (**D**), ii- rising levels of gamma-GT (from 33 to 392 U/L) (**E**) and iii- cumulative values of free-thyroxin (from 0.1 to 5.5 ng/mL) (**F**) and compared the findings to women at lower risks of breast cancer (L.Risk) as well as participants with stage III invasive disease (Breast Cancer). Results revealed that the same pattern generated by the ratio (Gln/Glu) when applied to cancer-free high risk participants (D), could be precisely recapitulated in blood of cancer-free women according progressive values of gamma-GT (E) and free-T4 (F). ^***^ Indicates *p* < 0.001 (H&N: Head and Neck Cancer).

To confirm these associations we conducted Pearson's r correlations (www.metaboanalyst.ca) that compared the described ratio values with levels of the oncometabolites fumarate, succinate, lactate, glutamine and hexoses [13, 14] measured in the blood of our 154 European breast cancer patients. The highest positive correlations were found with lactate (*p* = 1.42e-08, FDR = 3.24e-07), lactate/pyruvate (*p* = 7.96e-06, FDR = 7.47e-05) (Figure 3A) (Supplementary Table 1), fumarate/hexoses (*p* = 0.0004, FDR = 0.002), succinate/hexoses (*p* = 0.0001, FDR = 0.0007) and the glutaminolysis-related ratio (Ala+Asp+Glu/Gln) (*p* = 0.0004, FDR = 0.002) (Figure 1M). When the (Lac/Pyr) values were applied to the logistic regression equation logit(P) = log[P/(1–P)] = −12.24 + 1.80 Lac/Pyr, where P is Pr(y = 1|x), elevations in this ratio were associated with an increased risk of 5-years death (Odds = 6.08 [Pr (>IzI) = 0.001]) when analyzing patients with primary tumors not bigger than 2.0 cm (*n* = 103) (Supplementary Figure 1).

The highest negative correlations were observed for hexoses/lactate (*p* = 5.88e-08, FDR = 1.11e-06); hexoses (*p* = 0.002, FDR = 0.007); and the liver gluconeogenesis ratios (hexoses/PHGDH Act) (*p* = 0.002, FDR = 0.007); and (hexoses/Ala+Gly+Ser) (*p* = 0.0014, FDR = 0.005); [hexoses/(C14:1/C4)] (*p* = 0.003, FDR = 0.009); [hexoses/(C18:1/C8)] (*p* = 9.94e-05, FDR = 0.0006); (hexoses/CPTII) (*p* = 0.0007, FDR = 0.003); [hexoses/(C16/C3)] (*p* = 0.001, FDR = 0.004); (hexoses/AcylC-DC) (*p* = 0.002, FDR = 0.007) (Figure 1M) (Supplementary Table 2A, 2B).

### Correlations with other tumors of glandular ancestries

When the metabolic profiles of patients with different tumors (lung, colon, liver, leukemias, lymphomas and squamous cells carcinoma of head and neck) were examined, the results again demonstrated enhanced glutamine consumption, particularly in patients harboring tumors of glandular ancestries (Figure 2G).

Extending these studies to include patients with polycystic ovary syndrome (PCOS) (Black Arrow), cirrhosis (Blue Arrow), high-risk of breast cancer and stage 5 metabolic syndrome revealed that these cancer-free participants manifested glutaminolytic profiles that were very similar to those found in adenocarcinoma patients (Red) (Figure 2G).

The ratio (Glu/Hexoses) was assembled by us following the *in vitro* demonstration of the “glutamate pulling effect” (15) where glucose starvation in malignant cells culture leads to elevations in glutamate through a MYC-coordinated reaction.

This effect was clearly identified in the blood of patients harboring adenocarcinomas, those at higher risk of breast cancer (Red bar) and individuals with PCOS (Light orange bar) (Figure 3C). Noteworthy, neither of the control groups composed of population-based normal controls or patients with non-glandular tumors (leukemias, lymphomas, multiple myelomas and squamous cell carcinomas) revealed marked changes in this ratio particularly squamous cell carcinomas that revealed similar levels to controls (Figure 3C).

Increases in the “glutamate pulling effect” have been described under conditions of metabolic stress induced by glucose deprivation [15]. In agreement, we found a significant (*p* = 0.003, FDR = 0.009) inverse correlation between patient blood hexoses concentrations and the values of our breast cancer equation {[PC aa 36:6/[(Val/Phe)/Taurine]/C10:2} (Figure 1M).

**Figure 4:**
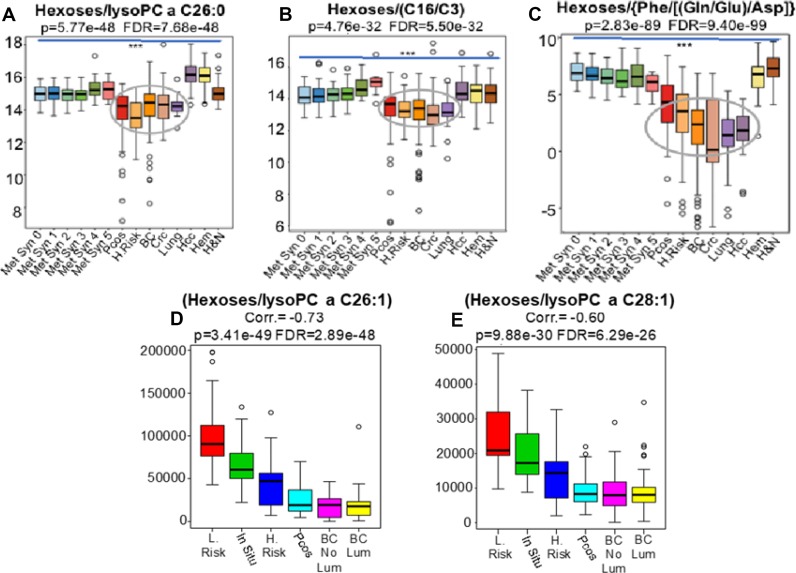
Our results suggest that the isolated determination of blood hexoses levels is not as informative as the measurement of hexoses levels in relation to other metabolic intermediates and ratios including: i) the mitochondrial carnitine palmitoyltransferase II (CPT II) deficiency ratio (C16/C3) (**B** Grey elipse) ii)-the peroxisomal impairment biomarkers lysoPC a C26:0, lysoPC a C26:1 and lysoPC a C28:1 (**A** Grey elipse) and **D**–**E** or iii)- its relation to glutaminolysis [Phe/(Gln/Glu)/Asp] (**C**). Importantly, both CPT II and peroxisomal deficiencies are well known metabolic conditions associated with hypoglycemia in patients afflicted with these rare metabolic disorders. BC, Breast Adenocarcinomas; CRC, Colon Adenocarcinomas; Lung, Lung Adenocarcinomas; HCC, Liver Adenocarcinomass; H.Risk (*n* = 33), women depicting 1.5 to 1.8 relative risks of breast cancer development; L.Risk, women at lower risks of breast cancer development; PCOS, Polycystic ovary syndrome; From 0 to 5, population-based controls depicting progressive stages of metabolic syndrome; Hem, patients harboring non-glandular tumors leukemias, myelomas and lymphomas; H&N, patients harboring squamous cells carcinomas of head and neck; BC-Lum (*n* = 104), patients harboring luminal breast tumors; BC-non Lum (*n* = 50), patients harboring non-luminal breast tumors; *In situ* (*n* = 23), patients harboring non-invasive *in situ* carcinoma ^***^ Indicates *p* < 0.001.

In line with the premise that glandular cancers are promoted under conditions of relative hypoglycemia, measured as the “glutamate pulling effect”, our results suggest that the isolated determination of blood glucose levels may not be as informative as the measurement of hexose levels in relation to other metabolic intermediates including: i) the mitochondrial carnitine palmitoyltransferase II (CPT-2) deficiency ratio (C16/C3) (Figure 4A) ii)-the peroxisomal impairment biomarkers lysoPC a C26:0, lysoPC a C26:1 and lysoPC a C28:1 (Figure 4B, 4D and 4E) or iii)- its relation to glutaminolysis [Phe/(Gln/Glu)/Asp] (Figure 4C). Importantly, both CPT-2 and peroxisomal deficiencies, well known inborn errors of metabolism, are associated with hypoglycemia in afflicted patients [16–18].

If a state of relative hypoglycemia were to occur in breast cancer as the result of inborn-like errors of metabolism then hyperinsulinemia associated with chronic hypoglycemia would constitute a powerful metabolic stressor capable of systemically up-regulating glycolysis and glutaminolysis, even in the absence of cancer.

### MYC-insulin hypoglycemic stress recapitulates biochemical disturbances associated with breast cancer

**Figure 5:**
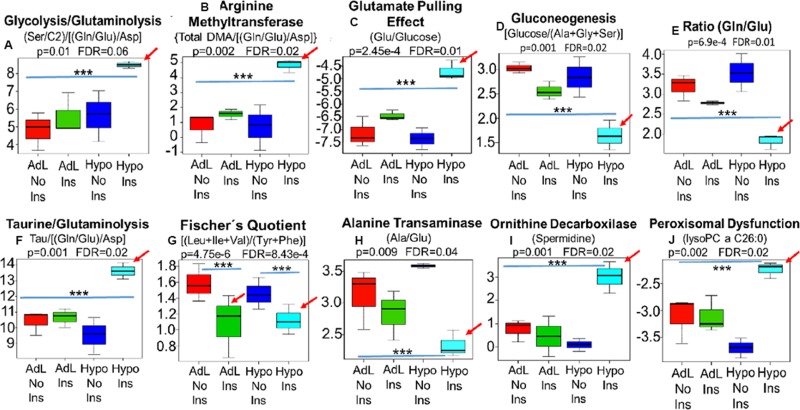
In this murine model, a proxy for human hyperinsulinemia/insulin resistance, only the hypoglycemic mice that received insulin (Hipo Ins) (Light blue bar) recapitulated the MYC-dependent shifts that had been observed in cancer patients, characterized by the insulin/MYC-dependent reactions of i: glutaminolysis and glycolysis (Gln/Glu), (Ser/C2)/[(Gln/Glu)/Asp] and (Ala/Glu) ii: glutamate pulling effect (Glu/Hexoses) iii: arginine methyltransferase activity [Total DMA/[(Gln/Glu)/Asp] and [Tau/[(Gln/Glu)/Asp] iiii: liver function [BCAA/(Phe+Tyr)] (Fischer's Quotient), ornithine decarboxylase activity (Spermidine), iiiiii: liver neoglucogenesis [Hexoses/(Ala+Gly+Ser)] and iiiiiii: peroxisomal impairment (lysoPC a C26:0) (**A**–**J**) (Red arrows). Notably, insulin was able to decrease liver function (Fischer) independent of glucose levels (G), however, decreases in ALT activity (H), neoglucogenesis (D) and peroxisomes function (J) were, exclusivelly seen in hypoglycemic mice that received insulin (Hipo Ins). AdL, ad libitum-feeded mice that did not receive insulin; AdL Ins, ad libitum-feeded mice that received insulin; Hipo No Ins, hypoglycemic mice that did not received insulin; Hipo Ins, hypoglycemic mice that received insulin; ^***^ Indicates *p* < 0.05.

To examine the hypoglycemia premise, we developed an experimental murine model in which insulin was administered to mice under normo- and hypoglycemic conditions [19, 20]. In this murine model only the hypoglycemic mice that received insulin (light blue) recapitulated the MYC-dependent shifts that had been observed in cancer patients, characterized by the insulin/MYC-dependent reactions of i: glutaminolysis (Gln/Glu), (Ala/Glu) and [(Gln/Glu)/Asp] as well as glycolysis (Ser/C2) and the combination of both (Ser/C2)/[(Gln/Glu)/Asp] ii: glutamate pulling effect (Glu/Hexoses) iii: arginine methyltransferase activity [Total DMA/[(Gln/Glu)/Asp] and [Tau/[(Gln/Glu)/Asp] iiii: liver function [BCAA/(Phe+Tyr)], ornithine decarboxylase activity (Spermidine), iiiiii: liver neoglucogenesis [Hexoses/(Ala+Gly+Ser)] and iiiiiii: peroxisomal impairment (lysoPC a C26:0) (Figure 5A–5J) (Red arrows).

To confirm these findings in humans, we examined whether blood concentrations of hexoses correlated with peroxisome dysfunction, as represented by the elevation of specific lipids containing very long chain fatty acids (VLCFA). We conducted “Pearson r” correlations to compare women at low risk of cancer (*n* = 31), to women at elevated relative risk (scoring 1.7 to 1.9) (*n* = 14), women with non-invasive (*in situ*) carcinoma (*n* = 23), women with polycystic ovary syndrome (*n* = 49) and those with invasive breast cancer both luminal (*n* = 118) and non-luminal (*n* = 36).

Results, from the ratios of hexoses to lysoPC a C26:1 (Correl. = −0.73, *p* = 3.41e-49, FDR = 2.89e-48) and hexoses to lysoPC a C28:1 (Correl. = −0.60, *p* = 9.88e-30 and FDR = 6.29e-29) demonstrated a progressive negative correlation beginning with women at high risk and *in situ* carcinoma, to PCOS and finally achieving a nadir in the plasma of patients with invasive disease, irrespective of intrinsic subtype (Figure 4D and 4E).

### Breast cancer as a consequence of a systemic, preexistent inborn-like error of metabolism

The results suggest that breast cancer could be preceded by systemic subclinical disturbances in glucose-insulin homeostasis characterized by mild, likely asymptomatic, IEM-like biochemical changes. The process would include variable periods of hyperinsulinemia with the consequent systemic MYC activation of glycolysis, glutaminolysis, structural lipidogenesis and further exacerbation of hypoglycemia, the result of MYC's known role as an inhibitor of liver gluconeogenesis [21].

Under normal conditions hypoglycemia results in the recruitment of fatty acids from storage pools. However, individuals who carry a primary inability to utilize fatty acids as an energy source, as seen in Fatty Acids Oxidation Defects (FAOD), would be prone to the accumulation of toxic oncometabolites as well as carnitine and fatty acid derivatives with increased ROS production and further mitochondrial disarrangement [22].

In this context, the metabolic dependencies of cancer characterized by excessive glycolysis, glutaminolysis and malignant lipidogenesis, previously considered a consequence of local tumor DNA aberration [23] could, instead, represent a systemic biochemical aberration that predates and very likely promotes tumorigenesis.

Furthermore these metabolic disturbances would be expected to remain extant after therapeutic interventions which is consistent with the recent observation that breast cancer relapse rates remain unaltered up to 24 years following initial treatments [24].

**Figure 6:**
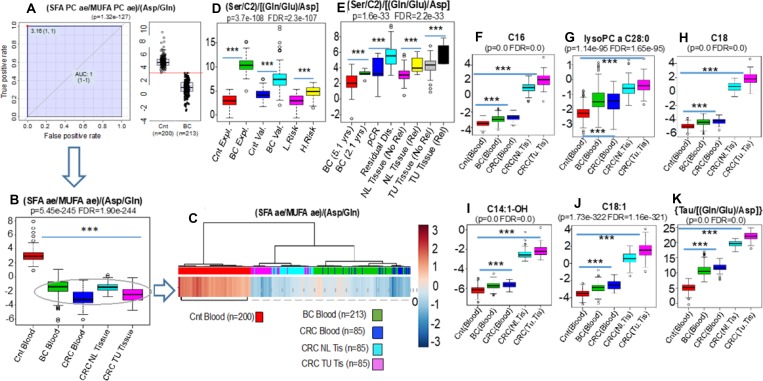
ANOVA and unsupervised clustering comparisons were assembled to compare the blood metabolic phenotypes from controls (Cnt blood *n* = 200), breast cancer (BC blood *n* = 213) (**A** and **B**) and colon cancer patients (*n* = 85) (CRC blood) with signatures obtained from both normal colonic epithelium (*n* = 85) (CRC NL Tissue) with respective colon cancer tissues (CRC TU Tissue) removed surgically from the same 85 CRC patients (B and **C**). These results demonstrate virtually identical biochemical phenotypes, evidenced by the ratio (SFA/MUFA)/(Asp/Gln), in the blood of breast (Green bar) and colon (Dark blue bar) cancer patients that are quantitatively indistinguishable from the phenotypic deviations detected in the normal colonic epithelium (Light Blue) and its respective colon tumor (Salmon) tissues (B and C). Interestingly, the biochemical disturbances found in both the normal (NL Tissue) and the tumor tissue (TU Tissue) samples from the same colon cancer patients and revealed by the ratio {(Ser/C2)/[(Gln/Glu)/Asp]}, additionally (*p* = 1.63e-33, FDR = 2.21e-33) identified those with the highest risk of relapse at 5 years (Rel) either after analyzing the normal (NL Tissue Rel) or tumor tissue samples (TU Tissue Rel) (**E**). This ratio in addition, clearly distinguished breast cancers from controls during exploratory (Expl) and validation (Val) sets as well as women at low (L.Risk) and high risk (RR = 1.8) (H.Risk) of cancer (**D**). In addition to the blood-based identification of cancer patients, the biochemical deviation identified by this same equation also distinguished i- women with shorter (BC 2.1 yrs) vs. longer (BC 5.1 yrs) relapse-free survival, and ii-women who achieved complete pathological response (pCR) vs. patients with residual disease (Residual Dis) after NAC (*p* = 3.73e-108, FDR = 2.31e-107) (E). Support for our hypothesis and consistent with the definition of IEM (18), we also detected the accumulation of very long chain acylcarnitines such as C14:1-OH (*p* = 0.0, FDR = 0.0), C16 (*p* = 0.0, FDR = 0.0), C18 (*p* = 0.0, FDR = 0.0) and C18:1 (*p* = 1.73e-322, FDR = 1.16-321) and lipids containing VLCFA (lysoPC a C28:0) (*p* = 1.14-e95, FDR = 1.65e-95) not only in the blood of breast and colon cancer patients but, importantly, in both the normal tissues and the tumor tissues from colon cancer patients (**F**–**K**). SFA, Sum of saturated acyl-alkyl phosphatidylcholines containing more than 36 carbons; MUFA, Sum of monounsaturated acyl-alkyl phosphatidylcholines containing more than 36 carbons.

In support for our hypothesis and consistent with the definition of IEM [22], we detected the accumulation of very long chain acylcarnitines such as C14:1-OH (*p* = 0.0, FDR = 0.0), C16 (*p* = 0.0, FDR = 0.0), C18 (*p* = 0.0, FDR = 0.0) and C18:1 (*p* = 1.73e-322, FDR = 1.16-321) and lipids containing VLCFA (lysoPC a C28:0) (*p* = 1.14-e95, FDR = 1.65e-95) in the blood of breast and colon cancer patients. Strikingly these same profiles were identified not only in the colon tumor tissues but also in the adjacent normal colonic mucosa removed at the time of surgery from these same colon cancer patients (Figure 6F–6K).

The metabolic changes we describe in breast cancer arise in concert with IEM-like changes in oxidative phosphorylation as detected by increased values of the ratio lactate/pyruvate (Supplementary Table 2A, 2B) characteristic of Ox/Phos deficiency [25]. In our study, 76% (70/92) of the European breast cancer patients had lactate/pyruvate ratios values higher than the normal value of 25.8.

Recent reports have identified a four-fold higher frequency of cancer (including breast) in patients with energy metabolism disorders [26] and IEMs are associated with elevated hexose/insulin disorders and gonadal and thyroid dysfunction that are themselves associated with high lactate/pyruvate ratios [18].

Defects in oxidative phosphorylation can occur as a result of primary fatty acid oxidation deficiencies (FAOD) as they are associated with the systemic mitochondrial accumulation of toxic fatty acid and carnitine derivative intermediates [27].

### Blood and normal tissues from cancer patients accumulate toxic metabolites that correlate with breast and colon cancer outcomes

To determine whether excessive glutaminolysis and glycolysis, as quantified in the current study, reflect systemic rather than local events, we hypothesized that the identified oncogenic disturbances should be present in the normal tissues, other than blood, of patients who harbor malignancies.

If true, then the biochemical profiles identified in these normal tissue biopsies should provide similar prognostic information with regard to response and survival to the data generated directly from tumor biopsy material.

Among the most powerful metabolic equations for MYC-activation is that which links the widely used MYC-driven desaturation marker ratio of SFA/MUFA to the MYC glutaminolysis-associated ratio of (Asp/Gln) [28, 29]. Our prior experience in 213 breast cancers and 200 controls revealed that the metabolic deviation underscored by this equation [(SFA/MUFA)/(Asp/Gln)], is one of the most robust breast cancer discriminants (AUC = 1.0, *p* = 1.32e-127) (Figure 6A and 6B).

ANOVA and unsupervised clustering comparisons were assembled to compare the blood metabolic phenotypes from controls (*n* = 200), breast cancer (*n* = 213) and colon cancer patients (*n* = 85) with signatures obtained from both normal colonic epithelium (*n* = 85) and colon cancers removed surgically from the same 85 CRC patients.

These results demonstrate virtually identical biochemical phenotypes, revealed by this equation in the blood of breast (Green bar) and colon (Dark blue bar) cancer patients that are quantitatively indistinguishable from the phenotypic deviations detected in the normal (Light Blue) and colon tumor (Salmon) tissues (Figure 6B and 6C). When compared with the control group (*n* = 200), the results from blood or tissue (both normal mucosa and tumoral) of the cancer patients are so concordant as to represent virtually indistinguishable biological samples.

Interestingly, the biochemical disturbances found in the normal colonic mucosa reflected in the ratio {(Ser/C2)/[(Gln/Glu)/Asp]}, significantly (*p* = 1.63e-33, FDR = 2.21e-33) correlated with the risk of relapse at 5 years indistinguishable from the results obtained with the colon tumors from these patients. (Figure 6E). This ratio not only clearly distinguished breast cancers from controls as well as women at low and high risk of cancer (Figure 6D) but also distinguished i- women with shorter (2.1 years) vs. longer (5.1 year) relapse-free survival, and ii-women who achieved complete pathological response (pCR) vs. patients with residual disease after NAC (*p* = 3.73e-108, FDR = 2.31e-107) (Figure 6E).

### Liver and thyroid dysfunctions are analogous to the metabolic disturbances seen in glandular malignancies

Additional observations in the present study found that liver dysfunction shares many features with both IEM and cancer suggesting a role for hepatic dysfunction in carcinogenesis.

Lower values of Fischer’s quotient [(Ile+Leu+Val)/(Tyr+Phe) (Figure 3A) and ALT activity (Ala/Glu) (Figure 3B), were found in cancer-free women with PCOS, those with elevated risks of cancer development and those with established glandular malignancies (liver, breast, colon, lung). These recurring biochemical deviations include transamination and gluconeogenesis frailties and the incapacity to properly metabolize branched chain (BCAA) and aromatic amino acids (Figure 3A and 3B).

The metabolic shifts evidenced by lower values in Fischer's ratio were not detected in any metabolic syndrome participant reflecting an accumulation of BCAA in blood, mainly in later stage disease wherein the Fischer's ratios were found to be higher. In adenocarcinoma patients the lower values of Fischer's ratio seem to reflect a deterioration of liver function resulting in a simultaneous diminution in BCAA and the accumulation of aromatic amino acids. Indeed, phenylalanine levels in breast cancer patients were found to be greater on average 89.3 μM/L (75 to 128 μM/L) than the normal expected values (40 to 74 μM/L) in 55% (85/154) of European breast cancer patients. Women scoring relative risks of 1.8 for breast cancer development also revealed elevated levels at 82.8 μM/L (64.6 to 98.8 μM/L) especially when compared to low risk women 70.3 μM/L (46.5 to 97.9 μM/L) and late stage metabolic syndrome with an average of 68 μM/L (47 to 95 μM/L). Patients with thyroid dysfunctions also exhibited higher levels of phenylalanine 94.6 μM/L (49.5 to 142 μM/L). As expected, cancer-free participants with cirrhosis exhibited the highest levels averaging 114.3 μM/L (84.4 to 163 μM/L).

To confirm these findings as liver-function related we included cancer-free patients with HCV-induced cirrhosis (*n* = 30) and patients with hypo (*n* = 8) and hyperthyroidism (*n* = 8), as thyroid dysfunction is frequently associated with liver dysfunction [30, 31] and with increased risk of cancer including breast [25]. We also analyzed HIV patients due to their increased risk of cancer and the direct effect of HIV infection on liver function [32].

Results revealed concordance between the blood metabolic profiles of cancer-free patients with cirrhosis, thyroid dysfunction and HIV infection and the study participants at: 1- elevated relative risks of breast cancer development, 2- those with PCOS and 3- patients harboring known glandular malignancies (breast, colon, lung and liver) (Figure 3A–3C).

We divided our cancer-free group according to: i-increasing risks of cancer, ii-rising levels of gamma-glutamyl transferase (GGT) and iii-cumulative values of free-thyroxine (Free T4). The results revealed the same pattern of Gln/Glu ratios when applied to high risk women, was recapitulated in cancer-free women by progressive changes in free-T4 and GGT values (Figure 3D–3F). Similar to thyroid dysfunctions [32], elevations in blood GGT have been found to significantly increase the overall cancer risk including breast malignancies [33]. To explore the biochemical overlap between these conditions we conducted Orthogonal Partial Least Squares Discriminative Analysis (Ortho-PLSDA) that revealed a high degree of biochemical similarity among hyper/hypothyroidism and cirrhosis patients that, together, seem to interconnect breast cancer on the one side to hematological malignancies on the opposite side. (Supplementary Figure 2).

It has previously been found that IEMs not only interfere with liver function but also affect proper endocrine physiology resulting in increased risks of diabetes, gonadal and thyroid dysfunctions [18].

As demonstrated in Figure 3A, 3B, 3D, and 3E, results identifying liver dysfunction are in agreement with the premise that breast cancer arises in an environment of fatty acid oxidation defects (FAOD). Among the most common laboratory findings in these types of IEM, in parallel with hypoglycemia, is liver dysfunction as the biochemistry of the liver is so dependent on the normal function of hepatocyte mitochondria [16].

Our findings, therefore, resemble those associated with mitochondrial and/or peroxisomal disorders of ß-oxidation, both known to be associated with the accumulation, in blood and tissues, of lipids composed of very long-chain fatty acids (VLCFA) and carnitine derivatives, the result of the inefficient oxidation of fatty acids [16].

In line with this concept, when controls (*n* = 92) were compared with breast cancer patients (*n* = 63) our untargeted mass spectrometry lipidomic data (Supplementary Figure 3A–3C) showed a global accumulation of phospholipid species containing very-long chain fatty acids (VLCFA ≥ C40) in the cancer patient specimens.

Of note are the blood elevations of lysoPC a C26:0, a biomarker routinely used in the diagnosis of peroxisomal disorders of ß-oxidation [34] which is identified by an arrow (Supplementary Figure 3A). Validation of this finding was subsequently obtained by specific targeted MS/MS (*p* = 9.07e-71, FDR = 2.81e-70) (Supplementary Figure 3B). Further suggestion of peroxisome as a putative subcellular location related to these metabolic findings, was obtained by quantitative functional enrichment analysis (www.metaboanalyst.ca) that revealed a significant (*p* = 1e-121) 250-fold enrichment for peroxisome localization using the metabolites L-acetylcarnitine, succinic acid, glycine, oxaloacetic acid, pyruvic acid, sarcosine, D-arginine and taurine (Supplementary Figure 4).

### Elevations in taurine and arginine methyltransferase activity are associated with breast cancer risk, response and survival

An additional finding was the significant elevations of taurine in the blood of breast cancer patients (Figure 1l) and its association with cancer risk, response and survival (Supplementary Figures 5 and 6) as well as its correlation with blood levels of the oncometabolites fumarate (*p* = 3.05e-06) and succinate (*p* = 1.87e-05) (Supplementary Table 2A and 2B).

Both fumarate and succinate are known to increase the half-life of HIF-1 gene (hypoxia-inducible factor-1) products that sponsor angiogenesis and tumor survival [35–38].

These oncometabolites also enhance histone and DNA methylation [39, 40] leading to genome-wide epigenetic reprogramming [41]. Taurine levels were also found to correlate (*p* = 0.001, FDR = 0.006) with the up-regulation of arginine methyltransferase activity, measured as the total amount of dymethylated arginine residues (Total DMA) (Supplementary Table 2A and 2B).

Total DMA levels were also gradually, positively and statistically (*p* = 5.57e-12, FDR = 1.56e-11) associated with progressive stages of breast carcinogenesis (Supplementary Figure 6C).

### Defining the cancer biochemistry as a fundamental “stemness” signature

Arginine methyltransferase activity is directly connected to MYC activity and has been reported to be associated to the state of cellular stemness [42–46].

This led us to question whether our breast cancer findings were reflective of a state of cellular biochemical stemness, as it has been suggested that there are considerable parallels between human embryogenesis and cancer [47–50].

To evaluate this hypothesis, we compared our breast cancer metabolomic signatures to those identified in the secretome of *in-vitro* fertilized, developing human embryos that were under final preparation for implantation (Supplementary Information)

Results demonstrated strong similarities between the metabolic profiles of successfully developed embryos and the biochemical phenotypes identified in women at high risk of breast cancer, those with insulin resistance and those with the shortest relapse-free survival following neoadjuvant chemotherapy. (Supplementary Figure 6D).

## DISCUSSION

We describe a new concept of carcinogenesis that incorporates our existing understanding of the genomic basis of cancer into a fundamentally different paradigm. Our findings suggest that cancer “conscripts” the human genome to meet its needs under conditions of systemic metabolic stress.

Health and cancer can be seen to reflect underlying IEM-like phenotypic states that result from variable levels of mitochondrial and peroxisomal dysfunction. These dysfunctions over the course of a normal lifespan might, or might not, lead to the condition of “metabolic insufficiency” that we recognize as cancer. As we age, the accumulation of toxic metabolites, onco-metabolites, DNA and histone methylation tips us from the state relative compensation to one of de-compensation as malignancy arises.

We describe blood biomarker panels based upon phenotypic features that are shared by IEM, liver and thyroid dysfunctions and cancers of glandular ancestries.

Using the identified signatures we explored correlations with other states of metabolic stress including diabetes mellitus and polycystic ovary syndrome and showed that we could recapitulate the malignant phenotype in a murine model by exposing hypoglycemic mice to exogenous insulin.

These phenotypic signatures share features of human cellular metabolic stemness and suggest that the same metabolic cascades that sponsor successful embryogenesis, a paradigm of stemness, are shared or re-activated, systemically, during periods of insulin/glucose imbalance.

The described metabolic stresses would, in the majority of the population, be counteracted by the up-regulation of gluconeogenesis and fatty acid oxidation. However, persons manifesting IEM-like phenotypes may be unable to marshal these critical responses, leading to the aberrant dependence upon MYC-related metabolic reprogramming.

This would reflect an underlying “tendency” to malignant transformation unleashed by stressors, that in breast cancer are “uncovered” by exacerbating risk factors, such as nulliparity, obesity and lifestyle but which only become manifest in those pre-disposed women who carry the features of inborn-priming.

The finding that the metabolic phenotype identified in the blood and tumor tissue of colon cancer patients is identical to the signature found in those same patients’ normal colonic mucosa supports our hypothesis that cancer arises as a local manifestation of a state a systemic metabolic insufficiency.

Variable levels of metabolic stress, therefore, would be different from individual to individual depending on inherited, mild to moderate metabolic deficiencies, reminiscent of IEM, but not severe enough to cause disease during much of life.

These signatures identify clinical breast cancer irrespective of stage, histology, intrinsic subtype, BMI, menopausal status or age with an accuracy of 95%, and are also shown to predict tumor response to neoadjuvant chemotherapy and overall survival.

There could be concern that the results reflect algorithms or ratios that were selectively defined to achieve desired results. We appreciate that concern and have made every effort to use training sets followed by confirmatory analyses and have applied well established biochemical parameters, previously described in the literature (Gln/Glu; Glutamate pulling effect, Fisher's quotient, etc.) in large data sets to statistically support our findings. We continue to analyze patients with breast cancer and other diseases both benign and malignant to further refine and confirm these observations.

The clinical implications of these findings are several and include the development of a new diagnostic test for the early detection of breast cancer and its application for prognosis and the prediction of response. The findings may also apply to other cancers of glandular histology. More importantly, the results reflect the application of a phenotypic signature that can dovetail nicely with advances in genomics, transcriptomics and proteomics as we strive for a more global understanding of human illness.

In conclusion, we provide phenotypic evidence to support the hypothesis that cancers of glandular ancestry, particularly breast cancer, represent the end result of pre-existing metabolic perturbations associated with a MYC-induced systemic condition: Cancer as a metabolic epiphenomenon.

## MATERIALS AND METHODS

### Nested case-control designs

All participants signed informed consent according to the applicable institutional human subject committee approvals including the Barretos Cancer Hospital (ethics approval CEP135/2008), São Paulo, Brazil; the Nutrition Department from the School of Public Health, University of São Paulo, Brazil; the Department of Obstetrics and Gynecology from the University of São Paulo; the Department of Obstetrics and Gynecology from the Medical University of Innsbruck in Austria; the Department of Gynecology from the Federal University of São Paulo, Brazil; the Department of Gynecology from the Hospital Meran in Meran, Italy and the Department of Gynecology from the Hospital Brixen, Brixen, Italy.

In total 1225 baseline samples were included being 1055 from blood and 170 from tissue specimens. Samples were prospectively collected from 2008 to 2011 and were analyzed by the same, fee-for-service, standardized, targeted quantitative mass spectrometry technique at the same centralized and independent company (Biocrates, Austria).

The cancer group (*n* = 473) were composed by: i-breast cancer volunteers from Brazil and Europe (*n* = 213) comprised in total by pT1pN0 (*n* = 68), pT1N1 (*n* = 77), pT2N1 (*n* = 8), T2N0M0 (*n* = 1) and T3N2M0 (*n* = 59). Intrinsic subtypes were: i-luminals A (*n* = 33), B (*n* = 98), B-HER2 (*n* = 23), triple negatives (*n* = 37), HER-2 (*n* = 14) and RE-/PR- (*n* = 4). European patients (*n* = 154) were composed by a retrospective (*n* = 62) and a prospective arm (*n* = 92) in addition to ii- lung (*n* = 23), iii- head and neck (*n* = 56), iiii-liver (*n* = 30), iiiii-hematological malignancies (*n* = 65) and iiiiii-colon cancer patients (*n* = 85) together to respective normal (*n* = 85) and tumor tissues (*n* = 85). Colon cancer patients were T1N0M0 (*n* = 9), T2N0M0 (*n* = 15), T3N0M0 (*n* = 20), T3N0M1 (*n* = 1), T3N1M0 (*n* = 10), T3N1M1 (*n* = 6), T3N2M0 (*n* = 6), T3N2M1 (*n* = 7), T4N0M0 (*n* = 2), T4N1M0(*n* = 1), T4N1M1 (*n* = 3), T4N2M0 (*n* = 2), T4N2M1 (*n* = 3).

The remaining 752 samples were included as control groups, out of which: 169 controls (79 women and 90 men) were from the São Paulo Population-based Health Investigation Project (ISA 2008) that due to its population characteristics, allowed us to analyzed them according their frequency of metabolic syndrome distributed according the 6 progressive stages following the recommendation of the Joint Interim Statement of the International Diabetes Federation Task Force on Epidemiology and Prevention; National Heart, Lung, and Blood Institute; American Heart Association; World Heart Federation; International Atherosclerosis Society; and International Association for the Study of Obesity [2].

Controls also included 33 women at elevated risks of breast cancer development (Suppl. Note), 23 participants with histologically proven non-invasive *in situ* carcinoma, 31 women at low risk of breast cancer development (Supplementary Note), 49 with polycystic ovary syndrome, 18 HIV–infected individuals prior of treatment, 34 women with rheumatoid arthritis, 58 autoimmune hemolytic disorders, 30 participants with cirrhosis, 8 with hyper and 8 with hypothyroidism.

Breast cancer patients with locally regional advanced tumors T3N2M0 (*n* = 59), were scheduled to receive a neoadjuvant chemotherapy approach comprised of 4 cycles of doxorubicin (60 mg/m^2^) and cyclophosphamide (600 mg/m^2^), followed by 4 cycles of paclitaxel (175 mg/m^2^) conducted at the Barretos Cancer Hospital, SP, Brazil.

This part of the study was designed to have as an endpoint the identification of predictive signatures of tumor response in patients with stage III disease, during the accomplishment of the project “Neoadjuvant Chemotherapy in Locally Advanced Breast Cancer (LABC)” (clinicaltrials NCT00820690). Patients, had a baseline assessment within 2 weeks before starting chemotherapy, hematological and non-hematological toxicities were recorded through complete blood counts, liver and kidney function as well as clinical evaluations at each cycle (one every 3 weeks time) and one month after the end of treatment.

Baseline tumor dimensions were calculated using clinical and radiological measurements and compared to the final tumor diameter that was recorded directly on the surgery product by a dedicated pathologist. Complete Pathologic Response (pCR) was defined as no histopathology evidence of any residual invasive and/or non-invasive disease in breast or nodes (ypT0/ypN0).

### Targeted quantitative MS/MS analysis

In this study, targeted metabolomic analysis of plasma and tissue samples was performed using the Biocrates Absolute-IDQ P180 (BIOCRATES, Life Science AG, Innsbruck, Austria). This validated targeted assay allows for simultaneous detection and quantification of metabolites in plasma and tissue samples in a high-throughput manner.

Absolute quantification (μmol/L) of blood metabolites was achieved by targeted quantitative profiling of 186 annotated metabolites by electrospray ionization (ESI) tandem mass spectrometry (MS/MS) in 1302 biological samples, blinded to any phenotype information, on a centralized, independent, fee-for-service basis at the quantitative metabolomics platform from BIOCRATES Life Sciences AG, Innsbruck, Austria.

The experimental metabolomics measurement technique is described in detail by patent US 2007/0004044 (accessible online at http://www.freepatentsonline.com/20070004044.html). Briefly, a targeted profiling scheme was used to quantitatively screen for fully annotated metabolites using multiple reaction monitoring, neutral loss and precursor ion scans. Quantification of metabolite concentrations and quality control assessment was performed with the MetIQ software package (BIOCRATES Life Sciences AG, Innsbruck, Austria) in conformance with 21CFR (Code of Federal Regulations) Part 11, which implies proof of reproducibility within a given error range. An xls file was then generated, which contained sample identification and 186 metabolite names and concentrations with the unit of μmol/L of plasma.

### Data analysis and validation tests

For metabolomic data analysis, log-transformation was applied to all quantified metabolites to normalize the concentration distributions and uploaded into the web-based analytical pipelines MetaboAnalyst 3.0 (www.metaboanalyst.ca/faces/upload/RocUploadView.xhtml) and Receiver Operating Characteristic Curve Explorer & Tester (ROCCET) available at http://www.roccet.ca/ROCCET for the generation of uni and multivariate Receiver Operating Characteristic (ROC) curves obtained through Support Vector Machine (SVM), Partial Least Squares-Discriminant Analysis (PLS-DA) and Random Forests as well as Logistic Regression Models to calculate Odds Ratios of specific metabolites [49–52].

ROC curves were generated by Monte-Carlo Cross Validation (MCCV) using balanced sub-sampling where two thirds (2/3) of the samples were used to evaluate the feature importance. Significant features were then used to build classification models, which were validated on the 1/3 of the samples that were left out on the first analysis. The same procedure was repeated 10-100 times to calculate the performance and confidence interval of each model.

To further validate the statistical significance of each model, ROC calculations included bootstrap 95% confidence intervals for the desired model specificity as well as accuracy after 1000 permutations and false discovery rates (FDR) calculation [49–52].

### Metabolite panel

In total, 186 annotated metabolites were quantified using the p180 kit (BIOCRATES Life Sciences AG, Innsbruck, Austria), being 40 acylcanitines (ACs), 21 amino acids (AAs), 19 biogenic amines (BA), sum of hexoses (Hex), 76 phosphatidylcholines (PCs), 14 lyso-phosphatidylcholines (LPCs) and 15 sphingomyelins (SMs). glycerophospholipids were further differentiated with respect to the presence of ester (a) and ether (e) bonds in the glycerol moiety, where two letters denote that two glycerol positions are bound to a fatty acid residue (aa = diacyl, ae = acyl-alkyl), while a single letter indicates the presence of a single fatty acid residue (a = acyl or e = alkyl). In the same company (Biocrates), the European participants had their samples additionally analyzed for the following energy metabolism metabolites: lactate, pyruvate/oxaloacetate, alpha ketoglutarate, fumarate and succinate.

### Metabolites ratios

In addition to individual metabolite quantification, groups of metabolites related to specific functions were assembled as ratios based on previous observation that the proportions between metabolite concentrations can strengthen the association signal and at the same time provide new information about possible metabolic pathways [53–58].

Additionaly, groups of AAs were computed by summing the levels of amino acids (AA) belonging to certain families or chemical structures depending on their functions such as the sum of: 1. essential amino acids (Essential AA), 2. non-essential amino acids (non-Essential AA), 3. glucogenic (Ala+Gly+Ser) amino acids (Gluc AA), 4. branched-chain (Leu+Ile+Val) amino acids (BCAA), 5. Aromatic (His+Tyr+Trp+Phe) amino acids (Arom AA), 6. Glutaminolytic derivatives (Ala+Asp+Glu) and the sum of total amino acids.

Groups of acylcarnitines (AC), important to evaluate mitochondrial function, were also computed by summing total acylcarnitines (AC), C2+C3, C16+C18, C16+C18:1 and C16-OH+C18:1-OH). Groups of lipids, important to evaluate lipid metabolism, were also analyzed by summing: 1. Total lysophosphatidylcholines (total LPC), 2. Total acyl-acyl and 3. Total acyl-alkyl phosphatidylcholines (total PC aa and total PC ae, respectively), 4. Total sphingomielins (total SM) and 5. Sum of total (LPC + PC aa +PC ae +SM) lipids (Structural lipids).

Proportions among sums of saturated, monounsaturated and polyunsaturated structural lipids were also assembled as proxies to estimate elongases and desaturases activities towards ether lipids: 1. Desaturase 9 [(PC ae C36:1 + PC ae C38:1 + PC ae C42:1) / (PC ae C42:0)], Desaturase 6 [(PC ae C44:6 + PC ae C44:5 + PC ae C42:5 + PC ae C40:6 + PC ae C40:5 + PC ae C38:6 + PC ae C38:5 + PC ae C36:5) / (PC ae C36:1 + PC ae C38:1 + PC ae C42:1)].

Clinical indicators of liver metabolism and function were obtained by applying either the classical (leucine+isoleucine+valine/(tyrosine+phenylalanine) or variations (Val/Phe, Xleu/Phe) of the Fischer's quotient. Clinical indicators of isovaleric acidemia, tyrosinemia, urea cycle deficiency and disorders of ß-oxidation were calculated by adopting the ratios of valerylcarnitine to butyrylcarnitine (C5/C4), tyrosine to serine (Tyr/Ser), glycine to alanine and glutamine (Gly/Ala, Gly/Gln) and lactate to pyruvate (Lac/Pyr), respectively [4, 30]. Proxies for enzyme function related to the diagnosis of very long-chain acyl-CoA dehydrogenase (VLCAD) and type 2 carnitine-palmitoyl transferase (CPT-2) deficiencies were achieved by assembling the ratios (C16+C18:1/C2), (C14:1/C4), (C14:1-OH/C9), (C14/C9), (C14:1/C9) and to the elongation of very-long-chain-fatty acids (ELOVL2) (PC aa C40:3/PC aa C42:5) [53–59].

Levels of methionine sulfoxide (Met-SO) alone or in combination to unmodified methionine (Met-SO/Met) as well as symmetric (SDMA), asymmetric (ADMA) and total dimethylation of argine residues (Total DMA) were quantified to gain access to ROS-mediated protein modifications as well as to systemic arginine methylation status [40, 41], respectively.

To gain access to MYC activity in blood, specific quantification of metabolites and ratios resulting from MYC-responsive enzymes activities were performed in the blood of hypoglycemic mice before and after insulin administration.

Knowing that liver inhibition of gluconeogenesis is a bona fide insulin-MYC-dependent biochemical reaction [17], a shift from normal to lower values in the ratio of glucose to glucogenic amino acids (Glucose/Ser, Glucose/Gly and Glucose/Ala) after insulin administration, was adopted as a measurement of insulin-MYC-related activity.

The same procedure was then applied to other MYC-responsive enzymes [42–44] as follows: arginine methyltransferases (ADMA, ADMA/Arg, SDMA, SDMA/Arg and Total DMA, Total DMA/Arg), ornithine decarboxylase (Glu, Glu/Orn, Pro, Pro/Orn, Orn, Orn/Arg, Putrescine, Putrescine/Orn, Spermidine, Spermidine/Putrescine, Spermine and Spermine/Spermidine), alanine aminotransferase (Ala), (Ala/Glu), aspartate aminotransferase (Asp) and (Asp/Glu), glutaminase (Glu), (Gln/Glu), [(Glu+Asp+Ala)/Gln], [(Gln/Glu)/Asp], (Glu/Glucose)/(Ala/Glu) and [(Glu/Gln)/Glucose]/(Ala/Glu).

The later 2 ratios were specifically assembled based on *in vitro* experiments related to the “glutamate pulling effect” which is defined as the hypoglycemia-induced up-regulation in the deaminated, rather than transaminated, production of glutamate through insulin-MYC-dependent glutamate dehydrogenase (GDH) stimulation of glutaminolysis with consequent increased amounts of net keto acids to anaplerosis [29].

We additionally included the ratios of (Ser/C2, Ser/Gln, Ser/Thr) and of (PC aa C42:0/PC ae C32:3, PC aa C32:2/PC ae C34:2) as proxies for glycolysis-related phosphoglycerate dehydrogenase (PHGDH) and glucokinase regulator (GCKR) activities [6–7].

The later inhibits glucokinase activity in liver and pancreas and the former catalyses the rate limiting step of serine biosynthesis [6–7].

In parallel, and assuming the ratio values of glutamine to glutamate (Gln/Glu) and to aspartate [(Gln/Glu)/Asp], as proxys for glutaminolytic activity, we finally assembled the ratios [(Ser/C2)/(Gln/Glu)], [(Ser/C2)/(Gln/Glu)/Asp], [(PC aa C32:2/PC ae C34:2)/(Gln/Glu)] and [(PC aa C32:2/PC ae C34:2)/(Gln/Glu)/Asp] as theoretical equations to gain access to the balance between glycolysis and glutaminolysis.

### Untargeted shotgun lipidomics MS/MS analysis

It was kindly performed by specialist personel (Helinho) in the AB-Sciex Laboratory located in Sao Paulo, SP, Brazil. Plasma samples from 59 breast cancer patients were compared to control group composed of 93 healthy menopaused volunteers (Supplementary Note). Samples were injected onto a Shimadzu Prominence LC system coupled to an AB-Sciex 5600 Triple TOF mass spectrometer instrument with an acquisition scan rate of 100 spectra/sec and stable mass accuracy of ~2 ppm.

Flow Injection Analysis (FIA) was performed using isocratic elution with Methanol/Water (90/10) with 5.0 mM of ammonium formate. Flow rate and injection volumes were 0.025 mL/min and 50 μL respectively.

No ion source or declustering potential (50 V and −40 V) optimization was performed. The following ionization parameters were applied: CUR = 20 psi, GS1 = 20 psi, GS2 = 15 psi, Temp = 250oC, IS = 5000 V (–4000V). MS scan ranging from m/z 100 to 1200 with accumulation time of 0.25 s and product ion scan from m/z 100 to 1200 and accumulation time of 0.03 s were the adopted parameters during survey and dependent scans respectively.

## SUPPLEMENTARY MATERIALS FIGURES AND TABLES






