# Supplementary material for: Inborn-like errors of metabolism are determinants of breast cancer risk, clinical response and survival: a study of human biochemical individuality
Source: Oncotarget. 2018 Aug 3;9(60):31664–81. doi: 10.18632/oncotarget.25839 (PMC6114970; doi:10.18632/oncotarget.25839)
Supplement: Supplementary file 1 [file oncotarget-09-31664-s001.pdf]

Inborn-like errors of metabolism are determinants of breast cancer risk, clinical response and survival: a study of human biochemical individuality

SUPPLEMENTARY MATERIALS

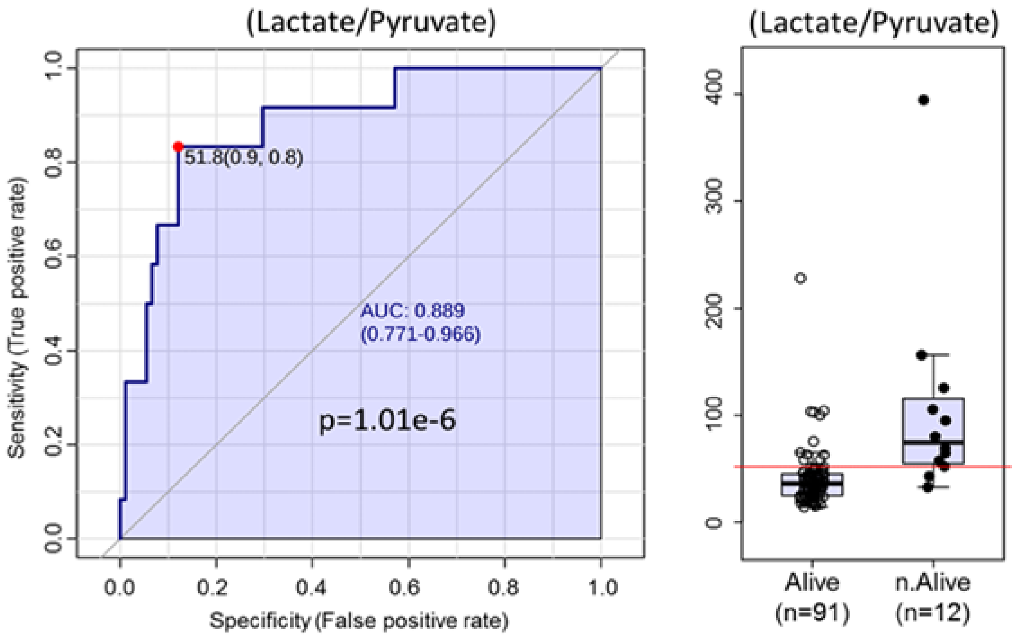

logit(P)= log[P / (1 - P)] = -12.24 + 1.80 Lac/Pyr, where P is Pr(y=1|x)

Logistic Regression Model	Estimate	Std. Error	z value	Pr(>|z|)	Odds
(Intercept)	-12.249	2.884	-4.248	< 0.001	*
Lac/Pyr	1.805	0.486	3.712	< 0.001	6.08

Sensitivity: 0.83 (0.62 - 1)
Specificity: 0.87 (0.82 - 0.93)
Positive Likelihood Ratio = 6.89
Negative Likelihood Ratio = 0.18

Supplementary Figure 1: Breast cancer cases (n = 103) from the European branch with primary tumors not bigger than 2.0 cm. The group of women that were dead within 5 years of follow-up were named n.Alive (n = 12) as opposed to the other group Alive (n = 91). The elevations of the ratio Lactate/Pyruvate (CutOff = 51.8) confered increased chances of death Odds = 6.08 [Pr (>IzI) = 0.001].

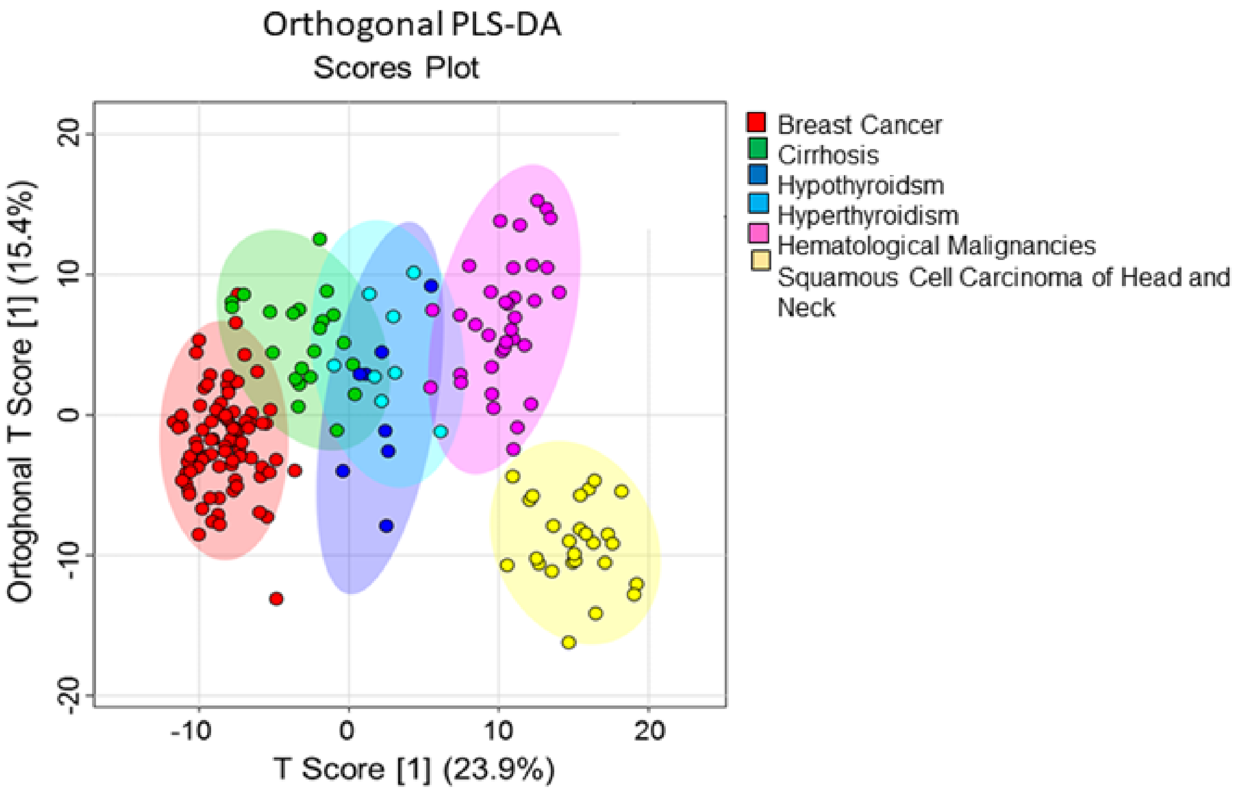

Empirical p-values R2Y: p < 0.001 (0/1000) and Q2: p < 0.001 (0/1000)

Supplementary Figure 2: Orthogonal partial least squares discriminative analysis (Ortho-PLSDA) was employed to simultaneously analyze the blood metabolic similarities among cancer-free patients with cirrhosis (green), hyper (light blue) and hypothyroidism (dark blue) with patients harboring breast cancer (red), squamous cells carcinoma (yellow) and hematological malignancies (salmon). The confidence intervals from each group are delimited by its corresponding fainted color, at their background, and can be used as a measurement of the biochemical overlapping among these conditions. Hyper and hypothyroidism in addition to liver dysfunctions share a high degree of metabolic similarities and, together, are able to interconnect breast cancer from one side to hematological malignancies from the other. Squamous cells carcinoma of head and neck, very likely due to its dependencies on external aggressions such as the ones inflicted by smoking, alcohol abuse and viral infections were grouped in a separated cluster.

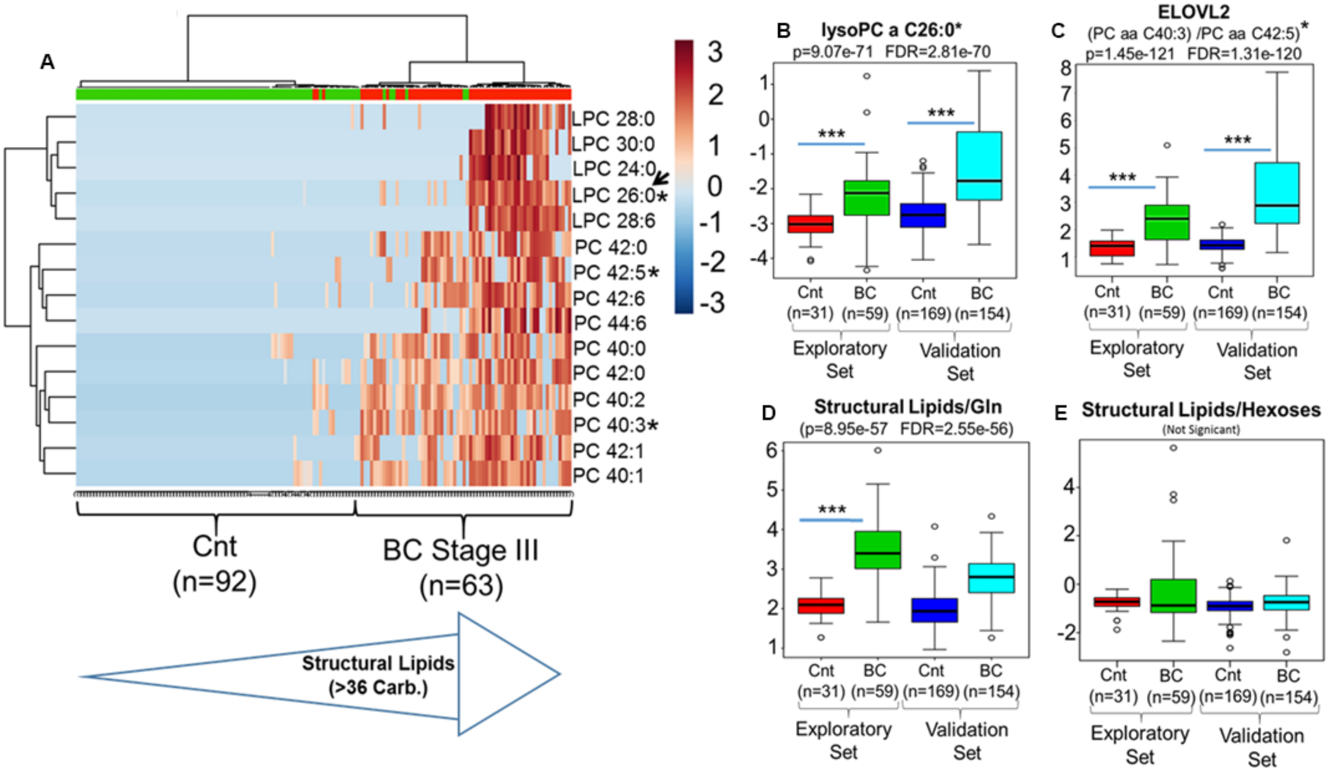

Supplementary Figure 3: Global accumulation of phospholipid species, containing very-long chain fatty acids (VLCFA), in the blood of breast cancer women harboring stage III invasive disease, pointed by an arrow, the increasing amounts of lysophosphatidylcholine C26:0 is considered a pathognomonic signal of peroxisomes dysfunction [29]. Validation of this finding was subsequently obtained by specific targeted MS/MS approach in the exploratory and validation sets (p = 9.07e-71, FDR = 2.81e-70) (**A**). Furthermore, since VLCFA higher levels are mainly the result of endogenous synthesis through elongation of long-chain fatty acids (60) we hypothesized that the biochemical pathways leading to lipid elongation would be favored in breast cancer women. Results depicted in (**C**) confirmed this possibility according to the ratio of PC aa C40:3/PC aa C42:5 as a proxy for the elongase of very long fatty acids 2 (eLOVL2) (3–7). The biochemical shift toward glutamine utilization, adopted by cancer cells during *in vitro* experiments (9), seems actually to be systemically active in the blood of breast cancer women as evidenced by the ratio (Structural lipids/Gln) (**D**) when compared to the ratio (Structural lipids/Hexoses) (**E**). Structural lipids, Sum of all Phospho and Sphingolipids; *Lipids utilized in (**B**) and (C). $^{***}p < 0.001$.

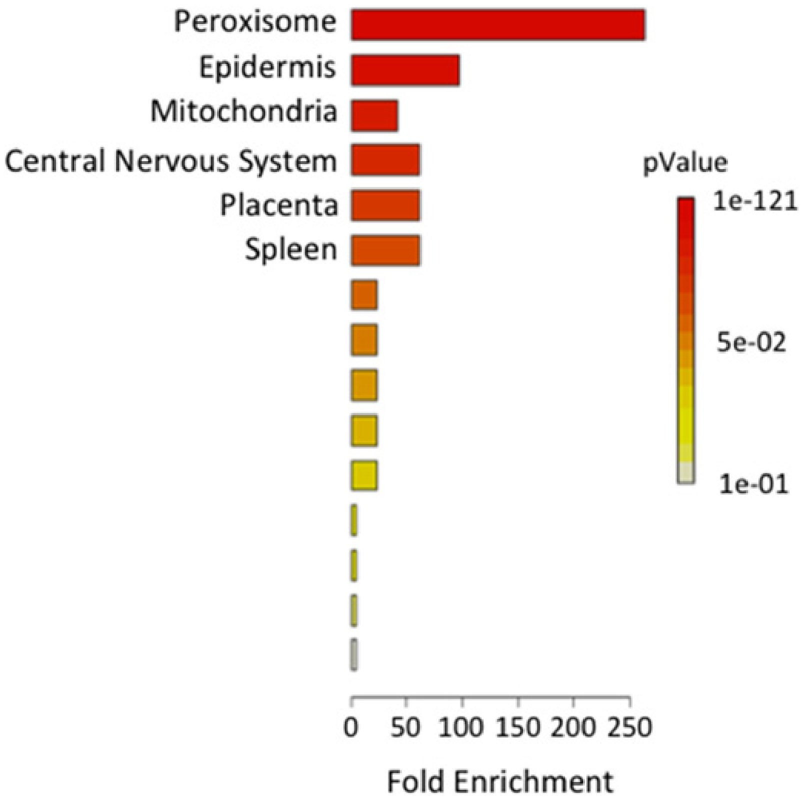

Supplementary Figure 4: Quantitative functional enrichment analysis after uploading the quantitative metabolite set from 154 breast cancer patients (Risk Prediction of Breast Cancer Metastasis Study) to the "Metabolite Set enrichment Analysis (MSeA)" tool available at www.metaboanalyst.ca. When interrogated for subcellular location, the quantitative results from L- acetylcarnitine, succinic acid, glycine, oxaloacetic acid, pyruvic acid, sarcosine, D-arginine and Taurine were considered for analysis and revealed a significant (*p* = 1e-121) 250-fold enrichment for peroxisomes.

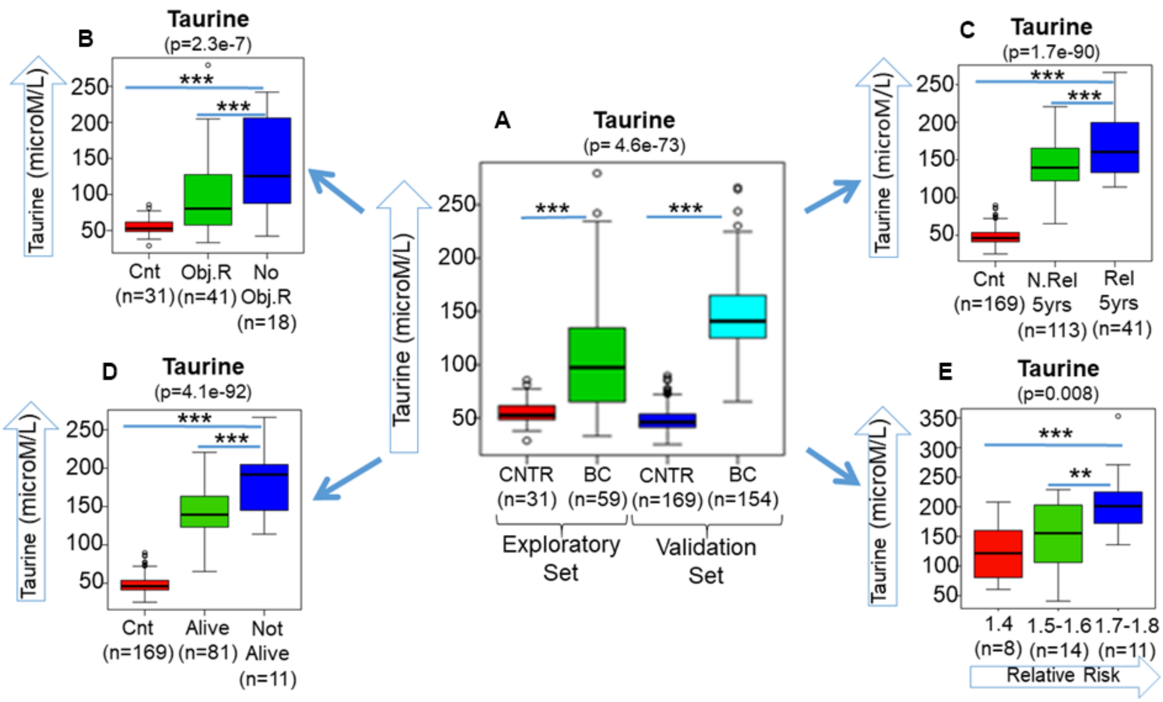

Supplementary Figure 5: Very likely because taurine and glycine conjugation to bile acids takes place at peroxisome level, another finding that could be related to peroxisome dysfunctions is the significant increased blood concentrations of taurine, detected at baseline of breast cancer women during training and validation sets (***p*** = 4.6e-73) (**A**) but also in women exhibiting no objective response (No Obj.) after neoadjuvant chemotherapy (*p* = 2.3e-7) (**B**), in addition to patients experiencing relapse (Rel) (*p* = 1.7e-90) (**C**) and death (Not Alive) within 5 years (*p* = 4.1e-92) (**D**) as well as in participants scoring elevated relative risks of breast cancer development (green and dark blue bars) (**E**) (*p* = 0.008). $^{***}p < 0.001$, $^{**}p < 0.01$ (RR = Relative Risk).

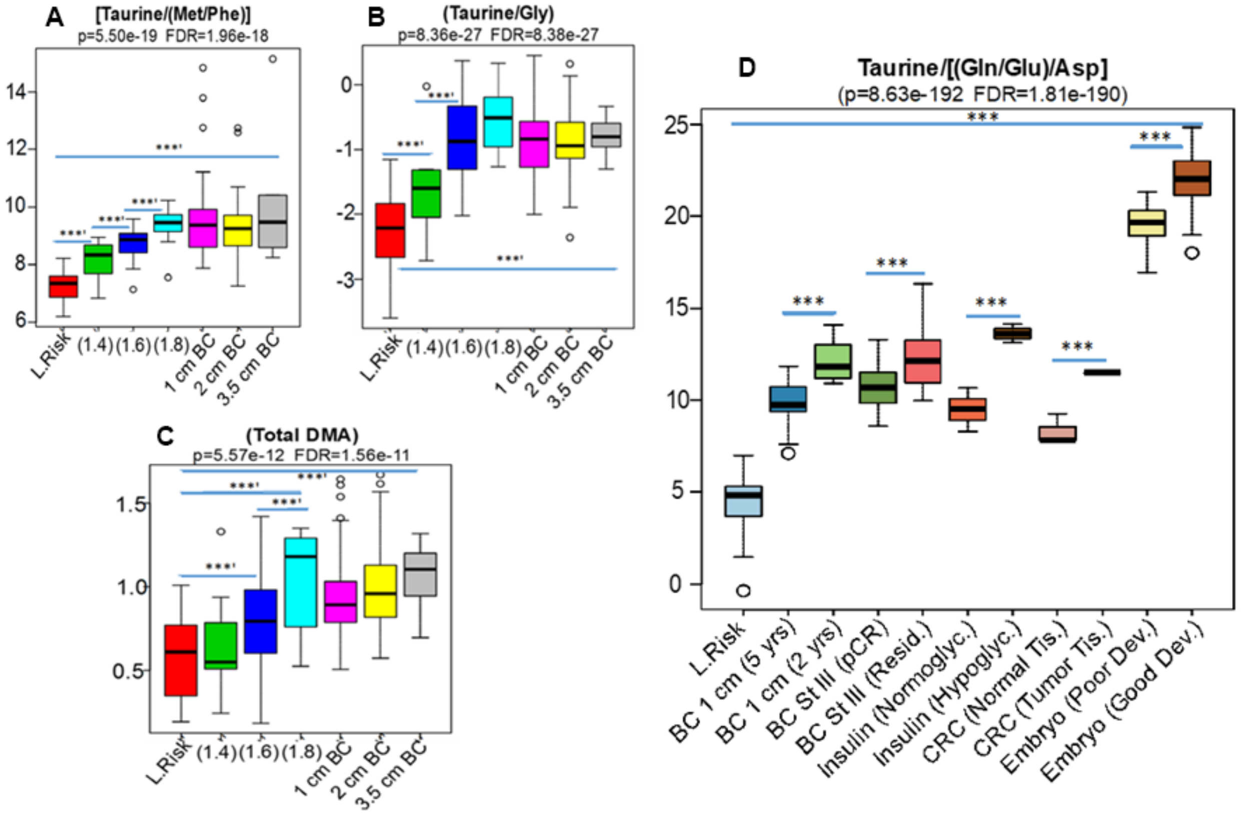

Supplementary Figure 6: Taurine is a methionine (Met) derivative and the ratio composed by [Taurine/(Met/Phe)], as a proxy for a product/substrate pair, is suggesting that taurine production seems to be proportional to increases in the relative risk of breast cancer development but once reaching invasive disease either with 1 cm or to 3.5 cm it seemed unchanged (**A**). Likewise, the ratio (Taurine/Gly) (**B**), largely used by bile acids researchers, seems to follow the same patern (B). Different from the previous ratios, the sum of symmetric and asymmetric dymethylated residues of arginine (Total DMA) (**C**) is gradually and positively associated to breast cancer progression is suggesting a direct correlation between increasing values and increasing tumor volumes (C). Of note, the proportions between taurine blood levels to glutaminolytic activity {Taurine/[(Gln/Glu)/Asp]} (**D**), besides association to chemotherapy response [BC Stage III (pCR)] versus [BC Stage III (Resid.)] and outcomes of survival (BC 1 cm 5 yrs) versus (BC 1 cm 2 yrs) were also found elevated in culture media where embryos of good development [embryo (Good Dev.)] were grown compared to culture media of low embryo development [embryo (Poor Dev.). Important, this very same ratio was found up-regulated in tumor tissues [CRC (Tumor Tis.)] compared to respective normal tissue counterparts [CRC (Normal Tis.)] from patients with colon cancer. Similar results were also obtained in hypoglycemic mice receiving insulin (Insulin Hypoglyc.) compared to normoglycemic animals (Insulin Normoglyc.) (RR = Relative Risk); [BC Stage III (Resid.)] = Stage III breast cancer with residual disease after neoadjuvant chemotherapy; [BC Stage III (pCR)] = Stage III breast cancer with complete pathological response after neoadjuvant chemotherapy; (BC 1 cm 5 yrs) = 1 cm tumors with overall survival >5 years; (BC 1 cm 2 yrs) = 1 cm tumors with overall survival <5 years. $^{***}p < 0.001$.

Supplementary Table 1(A–C): Identification and quantification of 186 metabolites in 7 classes of compounds using using the Absolute IDQ® p180 kit by Biocrates Life Sciences AG (Innsbruck, AT). See Supplementary_Table_1A–1C

Supplementary Table 2(A, B): Top metabolites positively (Salmon) and negatively (Blue) correlated to increasing values generated by the best breast cancer dicriminating ratio {PC aa C36:6/[(Xle/Phe)/Tau]}/C10:2. Results demonstrated significant positive correlations to oncometabolites such as lactate, succinate, fumarate and lactate/pyruvate in parallel to augments in lipids and acylcarnitines containing very long-chain fatty acids (C18:1, lysoPC a C28:0, lysoPC a C26:1) and increases in glutamate pulling effect (Glu/Glucose) that consequently induces a biosynthesis favored metabolic shift revealed by significant elevations in blood structural lipids (sum PCs and SMs). See Supplementary_Table_2A, 2B
