# Supplementary material for: Inborn-like errors of metabolism are determinants of breast cancer risk, clinical response and survival: a study of human biochemical individuality
Source: Oncotarget. 2018 Aug 3;9(60):31664–81. doi: 10.18632/oncotarget.25839 (PMC6114970; doi:10.18632/oncotarget.25839)
Supplement: Supplementary file 2 [file oncotarget-09-31664-s002.docx]

| Amino acids | |
| --- | --- |
| Code | Analyte |
| Ala | Alanine |
| Arg | Arginine |
| Asn | Asparagine |
| Asp | Aspartate |
| Cit | Citrulline |
| Gln | Glutamine |
| Glu | Glutamate |
| Gly | Glycine |
| His | Histidine |
| Ile | Isoleucine |
| Leu | Leucine |
| Lys | Lysine |
| Met | Methionine |
| Orn | Ornithine |
| Phe | Phenylalanine |
| Pro | Proline |
| Ser | Serine |
| Thr | Threonine |
| Trp | Tryptophan |
| Tyr | Tyrosine |
| Val | Valine |

| Biogenic Amines | |
| --- | --- |
| Code | Analyte |
| Ac-Orn | Acetylornithine |
| ADMA | Asymmetric dimethylarginine |
| SDMA | Symmetric dimethylarginine |
| alpha-AAA | alpha-Aminoadipic acid |
| Histamine | Histamine |
| Met-SO | Methionine-Sulfoxide |
| Kyn | Kynurenine |
| Putrescine | Putrescine |
| Sarcosine | Sarcosine |
| Spermidine | Spermidine |
| Spermine | Spermine |
| Serotonin | Serotonin |
| PEA | Phenylethylamine |
| Nitro-Tyr | Nitrotyrosine |
| c4-OH-Pro | cis-4-Hydroxyproline |
| t4-OH-Pro | trans-4-Hydroxyproline |
| Creatinine | Creatinine |
| Carnosine | Carnosine |
| Taurine | Taurine |
| DOPA | Dihydroxyphenylalanine |
| Dopamin | Dopamin |

| Acylcarnitines | |
| --- | --- |
| Code | Analyte |
| C0 | Carnitine (free) |
| C2 | Acetylcarnitine |
| C3 | Propionylcarnitine |
| C3:1 | Propenoylcarnitine |
| C3-OH | Hydroxypropionylcarnitine |
| C4 | Butyrylcarnitine / Isobutyrylcarnitine |
| C4:1 | Butenoylcarnitine |
| C4-OH (C3-DC) | Hydroxybutyrylcarnitine (Malonylcarnitine) |
| C5 | Isovalerylcarnitine / 2-Methylbutyrylcarnitine / Valerylcarnitine |
| C5:1 | Tiglylcarnitine / 3-Methyl-crotonylcarnitine |
| C5:1-DC | Glutaconylcarnitine / Mesaconylcarnitine |
| C5-DC (C6-OH) | Glutarylcarnitine (Hydroxyhexanoylcarnitine [= Hydroxycaproylcarnitine]) |
| C5-M-DC | Methylglutarylcarnitine |
| C5-OH (C3-DC-M) | Hydroxyisovalerylcarnitine / Hydroxy-2-methylbutyryl / Hydroxyvalerylcarnitine (Methylmalonylcarnitine) |
| C6 (C4:1-DC) | Hexanoylcarnitine [= Caproylcarnitine] (Fumarylcarnitine) |
| C6:1 | Hexenoylcarnitine |
| C7-DC | Pimelylcarnitine |
| C8 | Octanoylcarnitine [= Caprylylcarnitine] |
| C9 | Nonanoylcarnitine [= Pelargonylcarnitine] |
| C10 | Decanoylcarnitine [= Caprylcarnitine] |
| C10:1 | Decenoylcarnitine |
| C10:2 | Decadienoylcarnitine |
| C12 | Dodecanoylcarnitine [= Laurylcarnitine] |
| C12:1 | Dodecenoylcarnitine |
| C12-DC | Dodecanedioylcarnitine |
| C14 | Tetradecanoylcarnitine [= Myristylcarnitine] |
| C14:1 | Tetradecenoylcarnitine [= Myristoleylcarnitine] |
| C14:1-OH | Hydroxytetradecenoylcarnitine [= Hydroxymyristoleylcarnitine] |
| C14:2 | Tetradecadienoylcarnitine |
| C14:2-OH | Hydroxytetradecadienoylcarnitine |
| C16 | Hexadecanoylcarnitine [= Palmitoylcarnitine] |
| C16:1 | Hexadecenoylcarnitine [= Palmitoleylcarnitine] |
| C16:1-OH | Hydroxyhexadecenoylcarnitine [= Hydroxypalmitoleylcarnitine] |
| C16:2 | Hexadecadienoylcarnitine |
| C16:2-OH | Hydroxyhexadecadienoylcarnitine |
| C16-OH | Hydroxyhexadecanolycarnitine [= Hydroxypalmitoylcarnitine] |
| C18 | Octadecanoylcarnitine [= Stearylcarnitine] |
| C18:1 | Octadecenoylcarnitine [= Oleylcarnitine] |
| C18:1-OH | Hydroxyoctadecenoylcarnitine [= Hydroxyoleylcarnitine] |
| C18:2 | Octadecadienoylcarnitine [= Linoleylcarnitine] |

| Lysophosphatidylcholines | |
| --- | --- |
| Code | Analyte |
| lysoPC a C14:0 | Lysophosphatidylcholine with acyl residue C14:0 |
| lysoPC a C16:0 | Lysophosphatidylcholine with acyl residue C16:0 |
| lysoPC a C16:1 | Lysophosphatidylcholine with acyl residue C16:1 |
| lysoPC a C17:0 | Lysophosphatidylcholine with acyl residue C17:0 |
| lysoPC a C18:0 | Lysophosphatidylcholine with acyl residue C18:0 |
| lysoPC a C18:1 | Lysophosphatidylcholine with acyl residue C18:1 |
| lysoPC a C18:2 | Lysophosphatidylcholine with acyl residue C18:2 |
| lysoPC a C20:3 | Lysophosphatidylcholine with acyl residue C20:3 |
| lysoPC a C20:4 | Lysophosphatidylcholine with acyl residue C20:4 |
| lysoPC a C24:0 | Lysophosphatidylcholine with acyl residue C24:0 |
| lysoPC a C26:0 | Lysophosphatidylcholine with acyl residue C26:0 |
| lysoPC a C26:1 | Lysophosphatidylcholine with acyl residue C26:1 |
| lysoPC a C28:0 | Lysophosphatidylcholine with acyl residue C28:0 |
| lysoPC a C28:1 | Lysophosphatidylcholine with acyl residue C28:1 |

| Phosphatidylcholines - part1 | |
| --- | --- |
| Code | Analyte |
| PC aa C24:0 | Phosphatidylcholine with diacyl residue sum C24:0 |
| PC aa C26:0 | Phosphatidylcholine with diacyl residue sum C26:0 |
| PC aa C28:1 | Phosphatidylcholine with diacyl residue sum C28:1 |
| PC aa C30:0 | Phosphatidylcholine with diacyl residue sum C30:0 |
| PC aa C30:2 | Phosphatidylcholine with diacyl residue sum C30:2 |
| PC aa C32:0 | Phosphatidylcholine with diacyl residue sum C32:0 |
| PC aa C32:1 | Phosphatidylcholine with diacyl residue sum C32:1 |
| PC aa C32:2 | Phosphatidylcholine with diacyl residue sum C32:2 |
| PC aa C32:3 | Phosphatidylcholine with diacyl residue sum C32:3 |
| PC aa C34:1 | Phosphatidylcholine with diacyl residue sum C34:1 |
| PC aa C34:2 | Phosphatidylcholine with diacyl residue sum C34:2 |
| PC aa C34:3 | Phosphatidylcholine with diacyl residue sum C34:3 |
| PC aa C34:4 | Phosphatidylcholine with diacyl residue sum C34:4 |
| PC aa C36:0 | Phosphatidylcholine with diacyl residue sum C36:0 |
| PC aa C36:1 | Phosphatidylcholine with diacyl residue sum C36:1 |
| PC aa C36:2 | Phosphatidylcholine with diacyl residue sum C36:2 |
| PC aa C36:3 | Phosphatidylcholine with diacyl residue sum C36:3 |
| PC aa C36:4 | Phosphatidylcholine with diacyl residue sum C36:4 |
| PC aa C36:5 | Phosphatidylcholine with diacyl residue sum C36:5 |
| PC aa C36:6 | Phosphatidylcholine with diacyl residue sum C36:6 |
| PC aa C38:0 | Phosphatidylcholine with diacyl residue sum C38:0 |
| PC aa C38:1 | Phosphatidylcholine with diacyl residue sum C38:1 |
| PC aa C38:3 | Phosphatidylcholine with diacyl residue sum C38:3 |
| PC aa C38:4 | Phosphatidylcholine with diacyl residue sum C38:4 |
| PC aa C38:5 | Phosphatidylcholine with diacyl residue sum C38:5 |
| PC aa C38:6 | Phosphatidylcholine with diacyl residue sum C38:6 |
| PC aa C40:1 | Phosphatidylcholine with diacyl residue sum C40:1 |
| PC aa C40:2 | Phosphatidylcholine with diacyl residue sum C40:2 |
| PC aa C40:3 | Phosphatidylcholine with diacyl residue sum C40:3 |
| PC aa C40:4 | Phosphatidylcholine with diacyl residue sum C40:4 |
| PC aa C40:5 | Phosphatidylcholine with diacyl residue sum C40:5 |
| PC aa C40:6 | Phosphatidylcholine with diacyl residue sum C40:6 |
| PC aa C42:0 | Phosphatidylcholine with diacyl residue sum C42:0 |
| PC aa C42:1 | Phosphatidylcholine with diacyl residue sum C42:1 |
| PC aa C42:2 | Phosphatidylcholine with diacyl residue sum C42:2 |
| PC aa C42:4 | Phosphatidylcholine with diacyl residue sum C42:4 |
| PC aa C42:5 | Phosphatidylcholine with diacyl residue sum C42:5 |
| PC aa C42:6 | Phosphatidylcholine with diacyl residue sum C42:6 |

| Phosphatidylcholines - part2 | |
| --- | --- |
| Code | Analyte |
| PC ae C30:0 | Phosphatidylcholine with acyl-alkyl residue sum C30:0 |
| PC ae C30:1 | Phosphatidylcholine with acyl-alkyl residue sum C30:1 |
| PC ae C30:2 | Phosphatidylcholine with acyl-alkyl residue sum C30:2 |
| PC ae C32:1 | Phosphatidylcholine with acyl-alkyl residue sum C32:1 |
| PC ae C32:2 | Phosphatidylcholine with acyl-alkyl residue sum C32:2 |
| PC ae C34:0 | Phosphatidylcholine with acyl-alkyl residue sum C34:0 |
| PC ae C34:1 | Phosphatidylcholine with acyl-alkyl residue sum C34:1 |
| PC ae C34:2 | Phosphatidylcholine with acyl-alkyl residue sum C34:2 |
| PC ae C34:3 | Phosphatidylcholine with acyl-alkyl residue sum C34:3 |
| PC ae C36:0 | Phosphatidylcholine with acyl-alkyl residue sum C36:0 |
| PC ae C36:1 | Phosphatidylcholine with acyl-alkyl residue sum C36:1 |
| PC ae C36:2 | Phosphatidylcholine with acyl-alkyl residue sum C36:2 |
| PC ae C36:3 | Phosphatidylcholine with acyl-alkyl residue sum C36:3 |
| PC ae C36:4 | Phosphatidylcholine with acyl-alkyl residue sum C36:4 |
| PC ae C36:5 | Phosphatidylcholine with acyl-alkyl residue sum C36:5 |
| PC ae C38:0 | Phosphatidylcholine with acyl-alkyl residue sum C38:0 |
| PC ae C38:1 | Phosphatidylcholine with acyl-alkyl residue sum C38:1 |
| PC ae C38:2 | Phosphatidylcholine with acyl-alkyl residue sum C38:2 |
| PC ae C38:3 | Phosphatidylcholine with acyl-alkyl residue sum C38:3 |
| PC ae C38:4 | Phosphatidylcholine with acyl-alkyl residue sum C38:4 |
| PC ae C38:5 | Phosphatidylcholine with acyl-alkyl residue sum C38:5 |
| PC ae C38:6 | Phosphatidylcholine with acyl-alkyl residue sum C38:6 |
| PC ae C40:1 | Phosphatidylcholine with acyl-alkyl residue sum C40:1 |
| PC ae C40:2 | Phosphatidylcholine with acyl-alkyl residue sum C40:2 |
| PC ae C40:3 | Phosphatidylcholine with acyl-alkyl residue sum C40:3 |
| PC ae C40:4 | Phosphatidylcholine with acyl-alkyl residue sum C40:4 |
| PC ae C40:5 | Phosphatidylcholine with acyl-alkyl residue sum C40:5 |
| PC ae C40:6 | Phosphatidylcholine with acyl-alkyl residue sum C40:6 |
| PC ae C42:0 | Phosphatidylcholine with acyl-alkyl residue sum C42:0 |
| PC ae C42:1 | Phosphatidylcholine with acyl-alkyl residue sum C42:1 |
| PC ae C42:2 | Phosphatidylcholine with acyl-alkyl residue sum C42:2 |
| PC ae C42:3 | Phosphatidylcholine with acyl-alkyl residue sum C42:3 |
| PC ae C42:4 | Phosphatidylcholine with acyl-alkyl residue sum C42:4 |
| PC ae C42:5 | Phosphatidylcholine with acyl-alkyl residue sum C42:5 |
| PC ae C44:3 | Phosphatidylcholine with acyl-alkyl residue sum C44:3 |
| PC ae C44:4 | Phosphatidylcholine with acyl-alkyl residue sum C44:4 |
| PC ae C44:5 | Phosphatidylcholine with acyl-alkyl residue sum C44:5 |
| PC ae C44:6 | Phosphatidylcholine with acyl-alkyl residue sum C44:6 |

| Hexoses | |
| --- | --- |
| Code | Hexoses |
| H1 | Glucose |
| H1 | Aldohexose |
| H1 | L-Allopyranose |
| H1 | D-Allose |
| H1 | D-Allopyranose |
| H1 | D-Allose |
| H1 | D-Altropyranose |
| H1 | D-Glucopyranose |
| H1 | alpha-D-Glucopyranose |
| H1 | beta-D-Glucopyranose |
| H1 | D-Mannopyranose |
| H1 | alpha-D-Mannopyranose |
| H1 | L-Gulopyranose |
| H1 | D-Gulopyranose |
| H1 | D-Idopyranose |
| H1 | Alpha-L-Galactopyranose |
| H1 | alpha-D-Galactopyranose |
| H1 | beta-D-Galactopyranose |
| H1 | D-Talose |
| H1 | D-Talopyranose |
| H1 | Ketohexose |
| H1 | D-Psicopyranose |
| H1 | L-Fructofuranose |
| H1 | D-Fructose |
| H1 | D-Fructofuranose |
| H1 | L-Sorbopyranose |
| H1 | D-Sorbopyranose |
| H1 | D-Tagatose |
| H1 | D-Tagatopyranose |

| Sphingomyelins | |
| --- | --- |
| Code | Analyte |
| SM (OH) C14:1 | Hydroxysphingomyelin with acyl residue sum C14:1 |
| SM (OH) C16:1 | Hydroxysphingomyelin with acyl residue sum C16:1 |
| SM (OH) C22:1 | Hydroxysphingomyelin with acyl residue sum C22:1 |
| SM (OH) C22:2 | Hydroxysphingomyelin with acyl residue sum C22:2 |
| SM (OH) C24:1 | Hydroxysphingomyelin with acyl residue sum C24:1 |
| SM C16:0 | Sphingomyelin with acyl residue sum C16:0 |
| SM C16:1 | Sphingomyelin with acyl residue sum C16:1 |
| SM C18:0 | Sphingomyelin with acyl residue sum C18:0 |
| SM C18:1 | Sphingomyelin with acyl residue sum C18:1 |
| SM C20:2 | Sphingomyelin with acyl residue sum C20:2 |
| SM C22:3 | Sphingomyelin with acyl residue sum C22:3 |
| SM C24:0 | Sphingomyelin with acyl residue sum C24:0 |
| SM C24:1 | Sphingomyelin with acyl residue sum C24:1 |
| SM C26:0 | Sphingomyelin with acyl residue sum C26:0 |
| SM C26:1 | Sphingomyelin with acyl residue sum C26:1 |
