# Supplementary material for: Inborn-like errors of metabolism are determinants of breast cancer risk, clinical response and survival: a study of human biochemical individuality
Source: Oncotarget. 2018 Aug 3;9(60):31664–81. doi: 10.18632/oncotarget.25839 (PMC6114970; doi:10.18632/oncotarget.25839)
Supplement: Supplementary file 3 [file oncotarget-09-31664-s003.docx]

| **Metabolite** | **Correlation** | **T Stat** | **p-value** | **FDR** |
| --- | --- | --- | --- | --- |
| Lactate | 0.54939 | 6.2377 | 1.42E-08 | 3.24E-07 |
| Lactate/Pyruvate | 0.44694 | 4.7398 | 7.96E-06 | 7.47E-05 |
| Structural Lipids | 0.42469 | 4.4503 | 2.45E-05 | 0.00019926 |
| Spermidine | 0.4094 | 4.257 | 5.07E-05 | 0.00036409 |
| Succinate/Glucose | 0.38937 | 4.0104 | 0.00012493 | 0.00073749 |
| Glutamate/Glucose | 0.38023 | 3.9001 | 0.00018505 | 0.00099601 |
| lysoPC a C280 | 0.37721 | 3.864 | 0.00021014 | 0.0010987 |
| Fumarate/Glucose | 0.36046 | 3.6661 | 0.00041622 | 0.0020044 |
| Glutaminolysis | 0.35743 | 3.6308 | 0.00046909 | 0.0022297 |
| Glutamate/Glucose/ALT | 0.34739 | 3.5145 | 0.00069184 | 0.0031261 |
| CP 2 (C181/C8) | 0.32749 | 3.2882 | 0.0014395 | 0.0054237 |
| lysoPC a C261 | 0.31557 | 3.1549 | 0.002183 | 0.0076826 |
| Glutamate | 0.30453 | 3.0331 | 0.0031631 | 0.0099802 |
| Succinate | 0.3019 | 3.0043 | 0.0034478 | 0.010429 |
| Glucose/(C141/C4) | -0.30469 | -3.0348 | 0.0031464 | 0.0099802 |
| Glucose/PHGDH Act | -0.31593 | -3.159 | 0.0021559 | 0.0076608 |
| Glucose | -0.31593 | -3.159 | 0.0021559 | 0.0076608 |
| Glucose/Acyl DC | -0.31769 | -3.1786 | 0.0020292 | 0.0074269 |
| [Glucose/(Ala+Gly+Ser)] | -0.32751 | -3.2885 | 0.0014384 | 0.0054237 |
| [Glucose/(C16/C3)] | -0.33544 | -3.378 | 0.0010804 | 0.0043455 |
| (Glucose/CP 2) | -0.34707 | -3.5108 | 0.00070041 | 0.0031262 |
| (Gln/Glu) | -0.37602 | -3.8497 | 0.00022089 | 0.0011229 |
| Glucose/(C181/C8) | -0.39459 | -4.074 | 9.94E-05 | 0.00062694 |
| Glucose/Lactate | -0.52913 | -5.9157 | 5.88E-08 | 1.11E-06 |

Supplementary Table: 2A

Supplementary Table: 2B

Continuation

| **Metabolite** | **Correlation** | **T Stat** | **p-value** | **FDR** |
| --- | --- | --- | --- | --- |
| Lactate | 0.76 | 11.26 | 7.20e-19 | 6.57e-17 |
| [Methionine-Sulfoxide/(Hexoses/Lactate)] | 0.65526 | 8.22 | 1.37e-12 | 5.00e-11 |
| Aspartate | 0.62846 | 7.66 | 1.99e-11 | 6.60e-10 |
| (Lactate/Pyruvate) | 0.60577 | 7.22 | 1.58e-10 | 4.80e-09 |
| {Total Dimethylated Arginine/[(Gln/Glu)/Asp]} | 0.58992 | 6.93 | 6.10e-10 | 1.59e-08 |
| (Aspartate/Glutamine) | 0.52582 | 5.86 | 7.35e-08 | 1.49e-06 |
| (Fumarate/Hexoses) | 0.51632 | 5.71 | 1.38e-07 | 2.36e-06 |
| {Total Dimethylated Arginine/(Hexoses/Fumarate)] | 0.5098 | 5.62 | 2.10e-07 | 3.19e-06 |
| [Methionine-Sulfoxide/(Hexoses/Fumarate)] | 0.50521 | 5.55 | 2.81e-07 | 3.66e-06 |
| Ornithine transcarbamylase (Citruline/Ornithine) | 0.49694 | 5.43 | 4.69e-07 | 5.90e-06 |
| {Total Dimethylated Arginine/(Hexoses/Succinate)] | 0.49356 | 5.38 | 5.76e-07 | 6.78e-06 |
| Arginase 1 (Orn/Arg) | 0.49245 | 5.36 | 6.16e-07 | 7.03e-06 |
| Ornithine | 0.46987 | 5.04 | 2.30e-06 | 2.33e-05 |
| Fumarate | 0.46476 | 4.97 | 3.05e-06 | 2.93e-05 |
| Glutamate | 0.46037 | 4.91 | 3.89e-06 | 3.55e-05 |
| Spermidine | 0.45745 | 4.88 | 4.56e-06 | 4.06e-05 |
| (Succinate/Hexoses) | 0.45694 | 4.87 | 4.68e-06 | 4.07e-05 |
| [Methionine-Sulfoxide/(Hexoses/Succinate)] | 0.4524 | 4.81 | 5.97e-06 | 4.95e-05 |
| Succinate | 0.43019 | 4.52 | 1.87e-05 | 0.0001 |
| (Total Dimethylated Arginine/Hexoses) | 0.37402 | 3.82 | 0.0002 | 0.001 |
| (Methionine-Sulfoxide/Hexoses) | 0.33982 | 3.42 | 0.0009 | 0.004 |
| (Total Dimethylated Arginine/Arginine) | 0.32439 | 3.25 | 0.001 | 0.006 |
| Hexoses | -0.36229 | -3.68 | 0.0003 | 0.001 |
| (Hexoses/Succinate) | -0.49381 | -5.38 | 5.67e-07 | 6.78e-06 |
| (Hexoses/Fumarate) | -0.53.131 | -5.94 | 5.07e-08 | 1.09e-06 |
| (Hexoses/Lactate) | -0.7027 | -9.36 | 5.85e-15 | 2.67e-13 |
